# Stereocomplex-Driven Morphological Transition of Coil–Rod–Coil Poly(lactic acid)-Based Cylindrical Nanoparticles

**DOI:** 10.1021/acs.macromol.3c00653

**Published:** 2023-09-25

**Authors:** Yujie Xie, Wei Yu, Tianlai Xia, Rachel K. O’Reilly, Andrew P. Dove

**Affiliations:** † School of Chemistry, University of Birmingham, Edgbaston, Birmingham B15 2TT, U.K.; ‡ School of Medicine, Shanghai University, Shanghai 200444, China

## Abstract

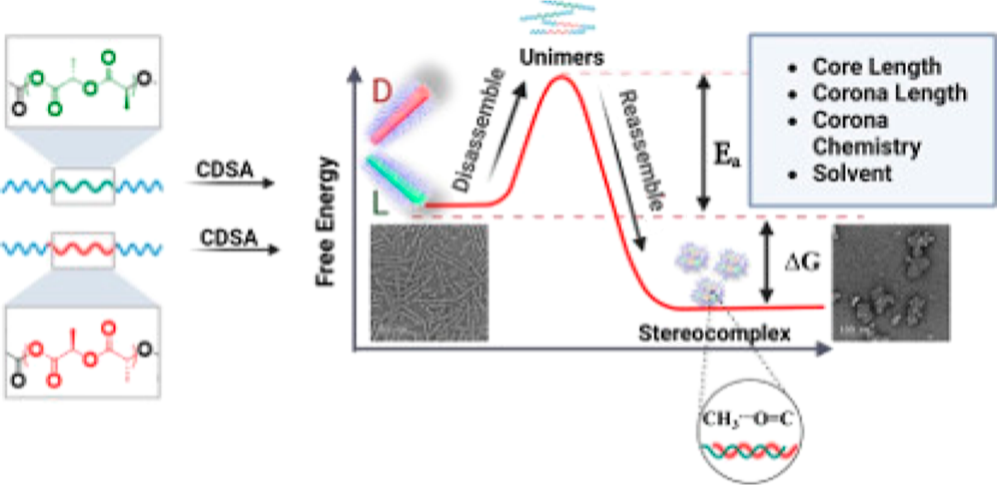

The stereocomplexation of poly(lactic acid) (PLA) enantiomers opens up an avenue for the formation of new materials with enhanced performance, specifically regarding their mechanical and thermal resistance and resistance to hydrolysis. Despite these useful features, the study of the stereocomplexation between block copolymers based on PLA in solution is limited, and a comprehensive understanding of this phenomenon is urgently needed. Herein, triblock copolymers of poly(*N*-hydroxyethyl acrylamide) and PL(or D)LA in which PLA was midblock (PHEAAm_*y*_-*b*-PL(D)LA_*x*_-*b*-PHEAAm_*y*_) were synthesized and assembled into cylindrical micelles via crystallization-driven self-assembly . The stereocomplexation between enantiomeric micelles facilitates the morphological transition, and the transformation process was investigated in detail by varying the aging temperature, block composition, and solvent. It was found that the solubility of the copolymers played a vital role in determining the occurrence and the speed of the chain exchange between the micelles and the unimers, which thereafter has a significant impact on the shape transition. These results lead to a deeper understanding of the stereocomplex-driven morphological transition process and provide valuable guidance for further optimization of the transition under physiological conditions as a new category of stimuli-responsive systems for biomedical applications.

## Introduction

Polylactide (PLA) is an aliphatic polyester that is biodegradable and biocompatible and displays low immunogenicity.^1–3^ While lactide exists as three stereoisomers (l-lactide, d-lactide, and *meso*-lactide), isotactic polymers formed by ring-opening polymerization of either l- or d-LA are the only ones that display crystallinity.^4^ Notably, when combined in equimolar quantities, poly(l-lactic acid) (PLLA) and poly(d-lactic acid) (PDLA) can cocrystallize to form a stereocomplex, which has better mechanical and thermal properties than those of their homochiral counterparts.^5,6^ The enhanced performance is a consequence of the thermodynamically favored CH_3_···O=C hydrogen bonding presented between the left-handed PLLA and right-handed PDLA polymeric helices and has been exploited as a strategy to access a variety of new materials with unique characteristics.^7–9^

Amphiphilic block copolymers are commonly designed to assemble into spherical and vesicular nanoparticles as drug-delivery vehicles in an aqueous solution. PLA-based block copolymer nanoparticles have been widely studied for these purposes; however, their stereochemistry is rarely exploited.^10^ Leroux and co-workers first reported stereocomplex block copolymer micelles in an aqueous solution by mixing equimolar quantities of enantiomeric PLLA-*b*-poly(ethylene oxide) (PLLA-*b*-PEO) and PDLA-*b*-PEO block copolymers.^11^ The stereocomplex micelles showed lower critical micellization concentration and higher kinetic stability than those of the equivalent enantiomeric pure micelles (i.e., PL(D)LA-*b*-PEO) on account of their more compact chain conformation in the core.^12–14^ Exploitation of stereocomplexation in PLA-based nanoparticles is not limited to coil-random structures. Other architectures such as Y-shaped miktoarm copolymers PEO–PL(D)LA_2_,^15^ coil-random-coil (poly(*N*-isopropylacrylamide)-PL(D)LA-poly(*N*-isopropylacrylamide)), random-coil-random (PL(D)LA–PEO-PL(D)LA) triblock copolymers,^16,17^ or bottle-brush polymers consisting of densely grafted PLLA and PDLA side chains^18^ are all able to form stereocomplex nanoparticles with lower critical aggregation/micelle concentration and enhanced stability over their homochiral counterparts in drug release tests. These approaches are therefore attractive methods to open new avenues to optimize nanocarriers for nanomedicine applications.

Micelles that have a cylindrical morphology have been demonstrated to have high potential as drug-delivery vehicles on account of the demonstration of prolonged in vivo circulation time and ability to display either active or passive targeting of desired tissues and organs.^19^ Recently, crystallization-driven self-assembly (CDSA) has emerged as a powerful tool to prepare cylindrical micelles with controllable lengths and high levels of uniformity.^20–27^ Previous work by both the groups of Manners and Winnik as well as ourselves has investigated the formation of cylindrical micelles by CDSA of enantiopure homochiral PLA-based diblock copolymers.^28,29^ Interestingly, we previously showed that by mixing cylinders with opposite homochirality (PLLA-*b*- PAA and PDLA-*b*-PAA), stereocomplexation drives a morphology change to the spheres that contain both PLLA and PDLA.^30,31^ It was proposed that the transition process followed a “unimer-exchange” mechanism when unimers were first dissolved from homochiral assemblies and then crystallized into new seeds from which stereocomplex particles grew.^32^ Notably, in this previous work, a high temperature (65 °C) and organic solvent (20% THF) were used to promote the transition.

**Scheme 1:**
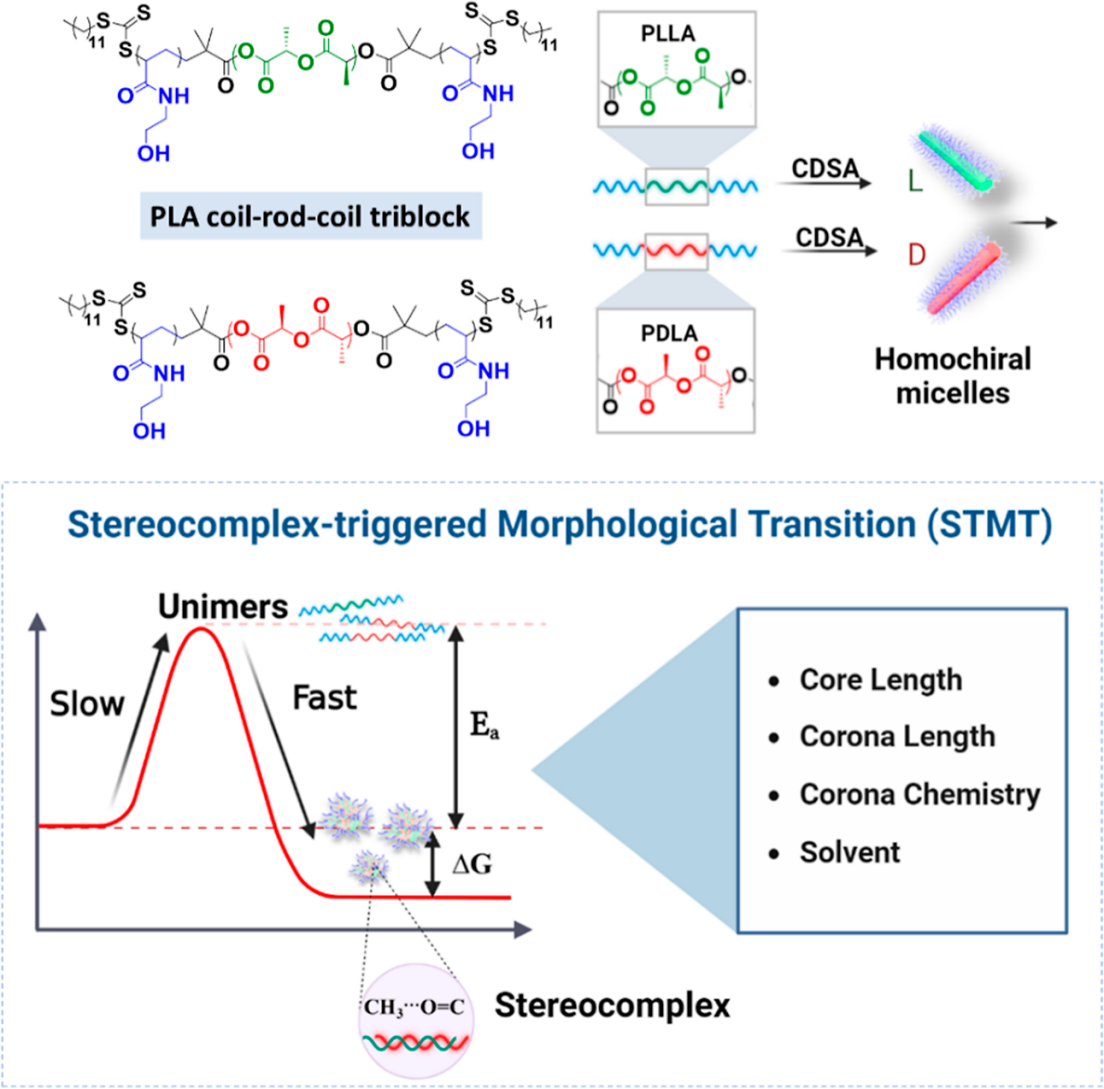
Process of Morphological Transition Triggered by Stereocomplexation between Polylactides

In order to enable the occurrence of the stereocomplex-driven morphology transition under more physiologically relevant conditions, we sought to improve the solubility of the hydrophilic block to facilitate the transition. Moreover, changing block copolymer architecture from diblock to triblock copolymers was also anticipated to further enhance the transition by lowering the energy barrier of the unimer extraction from the assemblies.^33^ To this end, we report the synthesis and application of a coil-rod-coil polymer architecture to prepare homochiral cylindrical PLA-based block copolymer nanoparticles using CDSA. Subsequently, the study of the stereocomplex-driven morphological cylinder-to-sphere transformation under mild conditions, e.g., physiological environment, was then conducted (Scheme 1). To enable this, the biocompatible and hydrophilic monomer *N*-hydroxyethyl acrylamide (HEAAm) was selected as the corona chemistry, and a series of triblock copolymers PHEAAm-*b*-PL(D)LA-*b*-PHEAAm were designed and assembled into cylindrical micelles. The effect of temperature, polymer composition, and solvent on the morphological transition process was investigated in detail, and these results paved the way to understand the stereocomplex-driven morphological transition process, such that it could be developed as a new stimuli-responsive system for biomedical applications.

## Results and Discussion

### Synthesis of ABA-Type Triblock Copolymers: PHEAAm_*y*_-*b*-PL(D)LA_*x*_-*b*-PHEAAm_*y*_

The triblock copolymers were synthesized by combining reversible addition–fragmentation chain-transfer polymerization (RAFT) and ring-opening polymerization (ROP) (Scheme S1) as described previously.^34^ 1,3-Propanediol was first used to initiate the polymerization of both d- and l-LA with 1,8-diazabicyclo(5.4.0)undec-7-ene (DBU) as an organic catalyst to yield the homochiral PLAs with no detectable racemization (Figure S1). The chirality of homochiral PLAs was further verified by optical rotation, which was determined to be [α]_D_^25^ = +130 and [α]_L_^25^ = −120 (*c* = 0.1 mg mL^–1^, CHCl_3_, 25 °C). After the successful coupling of the RAFT agent, DDMAT, to the polymer backbone using Steglich esterification (Figure S2), the coronal block was subsequently grown from the dual-headed macroCTAs through RAFT polymerization of HEAAm. The reaction was carried out in DMSO to minimize the hydrogen bonding between chains, and the conversion of the polymerization was controlled to reach a maximum of 70% in order to prevent termination and hence broadening the polymer dispersity. The composition of each block was calculated by proton nuclear magnetic resonance^(1^H NMR) spectroscopy using the known integral for the methine protons in the PLLA unit, and secondary amine signal corresponding to the HEAAm chemistry. Molecular weights and dispersities were characterized by size exclusion chromatography (SEC) analysis (Table 1, Figures S3 and S4). Importantly, all the triblock copolymers were shown to be monomodal with dispersity (*D̵*_M_) < 1.30.

**Table 1:** Characterization Data of PDMA-*b*-PL(D)LA-*b*-PDMA Triblock Copolymers

triblock copolymers	*M*_n_ (kDa)^a^	*M*_n_ (kDa)^b^	*D̵*_M_^b^	hydrophobic (% wt)^c^	no.
PHEAAm_42_-*b*-PLLA_32_-*b*-PHEAAm_42_, **L1**	14.3	20.1	1.21	32	**SA-L1**
PHEAAm_92_-*b*-PLLA_32_-*b*-PHEAAm_92_, **L2**	25.8	30.6	1.25	18	**SA-L2**
PHEAAm_65_-*b*-PLLA_50_-*b*-PHEAAm_65_, **L3**	22.2	28.5	1.19	33	**SA-L3**
PDMA_46_-*b*-PLLA_32_-*b*-PDMA_46_, **L4**	13.7	22.1	1.17	33	**SA-L4**
PHEAAm_50_-*b*-PDLA_32_-*b*-PHEAAm_50_, **D1**	16.1	21.3	1.16	29	**SA-D1**
PHEAAm_86_-*b*-PDLA_32_-*b*-PHEAAm_86_, **D2**	24.4	29.6	1.18	19	**SA-D2**
PHEAAm_70_-*b*-PDLA_50_-*b*-PHEAAm_70_, **D3**	23.3	28.7	1.21	30	**SA-D3**
PDMA_46_-*b*-PDLA_32_-*b*-PDMA_46_, **D4**	13.7	21.3	1.22	33	**SA-D4**

a Measured by ^1^H NMR (400 MHz, DMSO-*d*_6_).

b Measured by SEC (DMF with 5 mM NH_4_BF_4_).

c PLLA weight fraction in the PHEAAm_*y*_-*b*-PLLA_*x*_-*b*-PHEAAm_*y*_ triblock copolymers. All the assemblies were obtained by solubilizing (5 mg mL^–1^) and aging polymer in methanol at room temperature (25 °C).

### CDSA of PHEAAm_*y*_-*b*-PL(D)LA_*x*_-*b*-PHEAAm_*y*_ Triblock Copolymers

**Figure 1:**
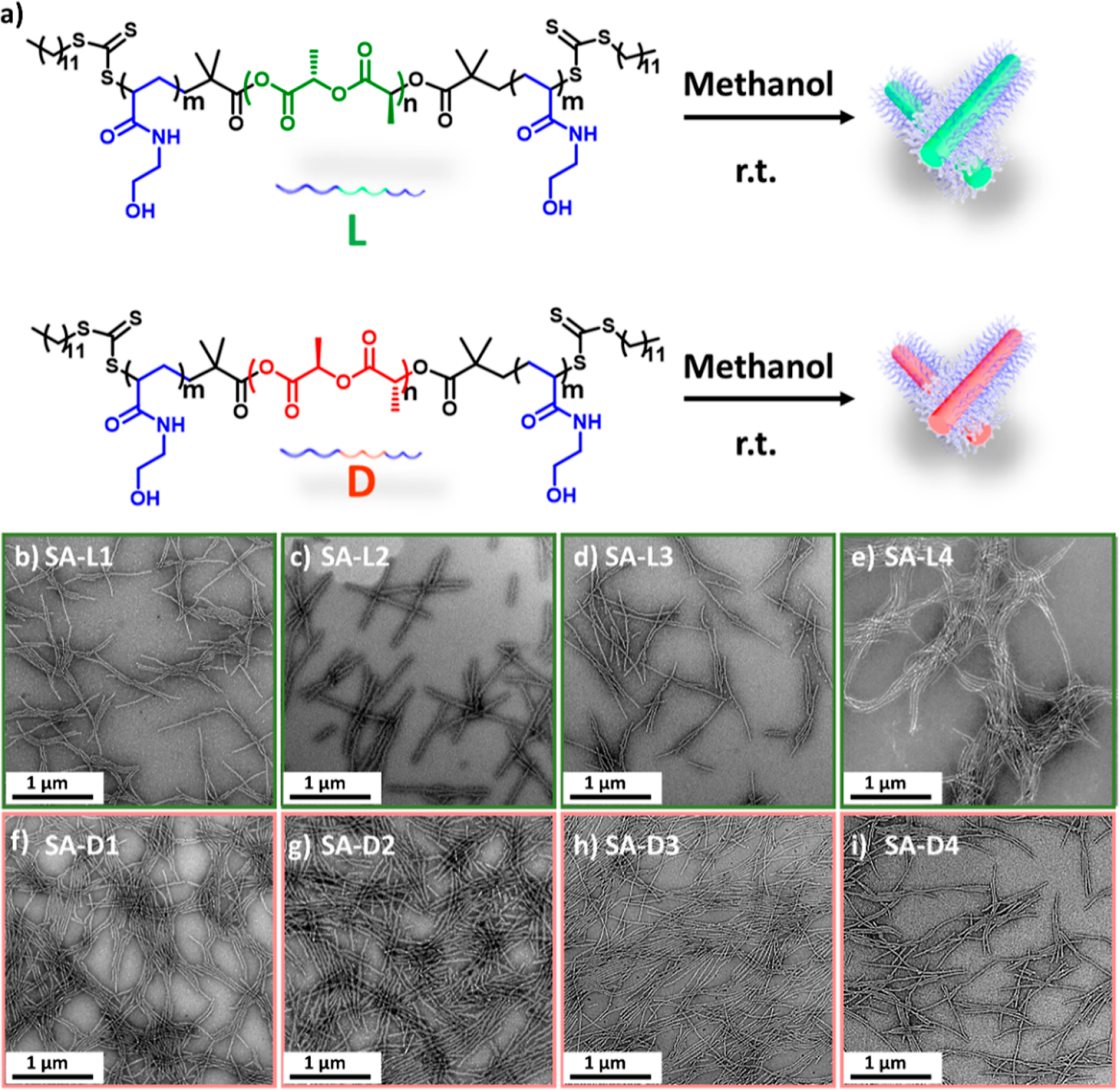
(a) Schematic illustration of the cylindrical micelle fabricated by the homochirality coil-rod-coil PLLA and PDLA triblock copolymers. (b–e) TEM micrographs of the PLLA copolymers assembled in methanol 5 mg mL^–1^ at room temperature (25 °C) for 2 days: (b) **SA-L1**, (c) **SA-L2**, (d) **SA-L3**, and (e) **SA-L4**. (f–i) TEM micrographs of PDLA copolymers assembled in methanol 5 mg mL^–1^ at room temperature (25 °C) for 2 days: (f) **SA-D1**, (i) **SA-D2**, (g) **SA-D3**, and (h) **SA-D4**. All TEM samples were negatively stained using uranyl acetate (0.5 wt %). Scale bar = 1 μm.

In order to identify an optimal solvent to undertake CDSA, singular alcoholic solvents (e.g., methanol or ethanol) were used to prepare 1D micelles of stereoregular PLA-based block copolymers via CDSA in a similar manner to that reported previously.^35,36^ In short, this involved dissolution and aging of the triblock copolymer in methanol at a concentration of 5 mg mL^–1^ at room temperature (Figure 1a).^34^ After dissolution in methanol aided by vortexing and sonication, a weak Tyndall effect was observed after only 1 h. After aging for 24 h, the solution became turbid and displayed a significant Tyndall effect (Figure S5), which indicates that larger micelles had formed (**SA**-**L1** to **SA-D4** in Table 1).

Investigation of the dimensions and shape of the nanoparticles formed was performed by transmission electron microscopy (TEM) analysis. Polymer **L1** (with a PLLA midblock) assembled into cylindrical micelles (**SA-L1**) with length values ranging from 200 nm to 2 μm, while the diameter was ca. 30 nm (Figure 1b). When the corona-to-core ratio was increased from 2:1 to 4:1 (**L2**), the width of the resulting cylinders also increased to 40 nm (Figure 1c). In contrast, the increase in core length (from degree of polymerisation (DP) = 32 to DP = 50 with the same corona-to-core ratio) had no effect on the assembly morphology in which well-defined fiber-like nanoparticles were achieved with similar width (Figure 1d,e). The results obtained for the enantiomer assemblies of copolymer PHEAAm_*y*_-*b*-PDLA_*x*_-*b*-PHEAAm_*y*_ (Figure 1f–i) were in good agreement with those obtained for their PLLA counterpart. Hence, in general, cylindrical micelles were achieved from PHEAAm_*y*_-*b*-PL(D)LA_*x*_-*b*-PHEAAm_*y*_ triblock copolymers following this simple approach, regardless of their block ratio (corona-to-core ratio varied from 4:1 to 2:1) or, as may be anticipated, the chirality of the PLA block. To confirm the stability of the assemblies in methanol, the nanoparticles were aged for 1 month at room temperature. In all cases, little change in the morphology was observed. For instance, assemblies **SA-L1** and **SA-L2** displayed negligible morphological differences (Figure S6) in comparison to the original morphology (aging 2 days, Figure 1b,c), which evidence the robustness of the self-assembled cylindrical micelles. Finally, to exclude the possibility of the observed cylinders being artifacts that occurred as a result of staining in the TEM technique, samples, e.g., **SA-L1** and **SA-D1**, were also characterized by atomic force microscopy (AFM), which confirms that the shape and size of the nanoparticles (Figure S7) are the same as observed by TEM analysis.

### Stereocomplexation-Driven Morphological Transition in Methanol: From Homochiral Cylinders to Stereoaggregates

The morphological cylinder-to-sphere transition, driven by stereocomplexation of the PLLA core, was subsequently investigated by mixing equal amounts of opposite enantiomer cylindrical assemblies (**SA-L1** and **SA-D1**). While our previous study into this phenomenon was conducted at 60 °C in H_2_O/THF, we wanted to move toward physiological conditions. To this end, we studied the stereocomplexation-driven morphological transition (SDMT) that occurred in methanol (1 mg mL^–1^) at body temperature (i.e., 37 °C) after aging for 24 h. Surprisingly, after that period, few cylindrical micelles were observed on the TEM grid, whereas a new aggregated sphere-like structure had appeared (Figure S8).

**Figure 2:**
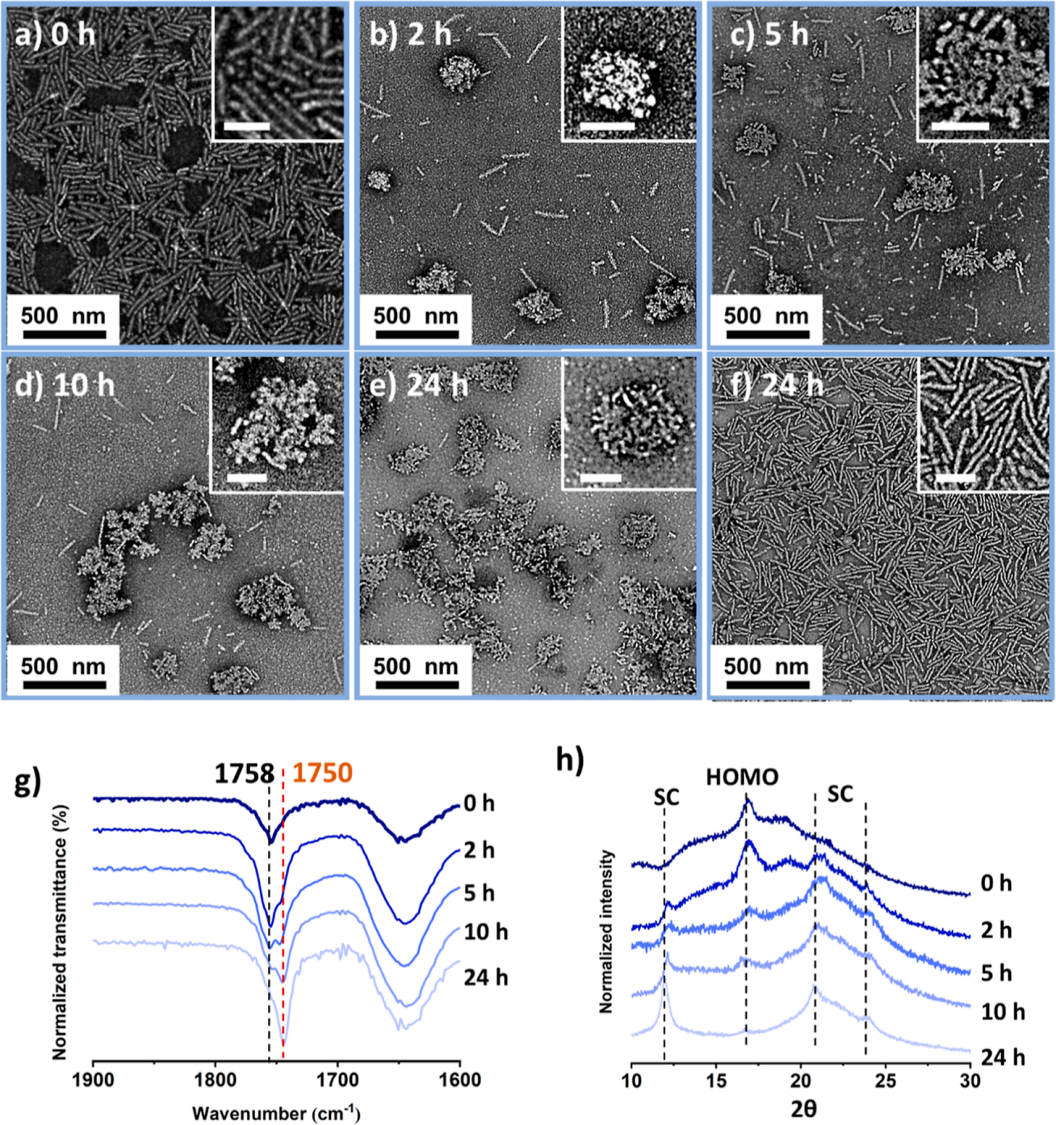
TEM micrographs of the mixed assembly solution **SA-L1** and **SA-D1** after aging at 37 °C for (a) 0, (b) 2, (c) 5, (d) 8, and (e) 24 h. (f) Homochiral micelles **SA-L1** aged at body temperature (37 °C) for 24 h. The samples were negatively stained using uranyl acetate (0.5 wt %). (g) FTIR spectra revealed that the wavenumber of the carbonyl group vibration of PLA shifted from 1758 to 1750 cm^–1^ over time during the morphological transition process. (h) Wide-angle X-ray diffraction analysis of the sample at 0, 5, and 24 h.

To understand this transition process, a kinetic study was conducted. The long cylindrical micelles (**SA-L1** and **SA-D1**) were sonicated separately into shorter cylinders (*L*_n_ = 50–300 nm, Figure S9) to obtain comparatively uniform micelles. After sonication, both solutions were mixed in methanol and aged at 37 °C, and aliquots were taken at 2, 5, 10, and 24 h. Interestingly, the formation of stereocomplex structures was detected after aging for only 2 h (Figure 2b), which indicated that the initiation of the morphological transformation was rapid. As the aging time evolved, the number of cylinders observed on the grid gradually decreased, while the newly formed morphology increased accordingly. After 24 h, fiber-like micelles were hardly detected, which suggested that the morphological transition was almost finalized. A control experiment was performed by aging the pure chiral micelles (**SA-L1**) in the same conditions (methanol, 37 °C) for 24 h while the micelles remained in the original state, i.e., cylindrical morphology (Figure 2f).

To further confirm that the observed morphological transition resulted from the stereocomplexation of the PLA blocks, Fourier-transformed infrared (FTIR) spectroscopic analysis was performed on the micelles. FTIR is able to confirm the formation of stereocomplex lactide enantiomer as a result of the vibrational wavenumber of the carboxyl functional group shifting from 1758 cm^–1^ for the homochiral PLA to 1750 cm^–1^ for the stereocomplex PLA.^32^ Following deconvolution of the vibrational peaks of the FTIR spectra between 1720 and 1780 cm^–1^ over the course of the 24 h study (Figure S10), a clear shift of the carbonyl vibration was observed, which confirms the evolution of PLA from a homochiral polymer to a stereocomplex (Figure 2g). Specifically, the progression of the transformation (*P*) was defined by the area integration percentage of the signal corresponding to 1750 cm^–1^. Although there are some limitations to predict the transition process using this approach, the data collected and analyzed under the same condition within this system are still informative to compare and draw conclusions.
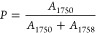
1

Noticeably, after 2 h, the progression of the transformation reached 41% (Figure 2g). Moreover, to corroborate the formation of the stereocomplex micelles, the assembled samples were prepared for wide-angle X-ray scattering (WAXS) analysis. The presence of sharp Bragg peaks at a 2θ value of 12 and 23.8°, which correspond to the stereocomplex of PLA, further confirmed the formation of a new crystalline structure gradually (Figure 2h). Meanwhile, the Bragg peak attributed to the homochiral polymer at a 2θ value of 16.6° decreased accordingly, confirming the transition from homochiral to stereocomplex micelles in 24 h.

### Elucidating the Mechanism of Stereocomplexation

**Figure 3:**
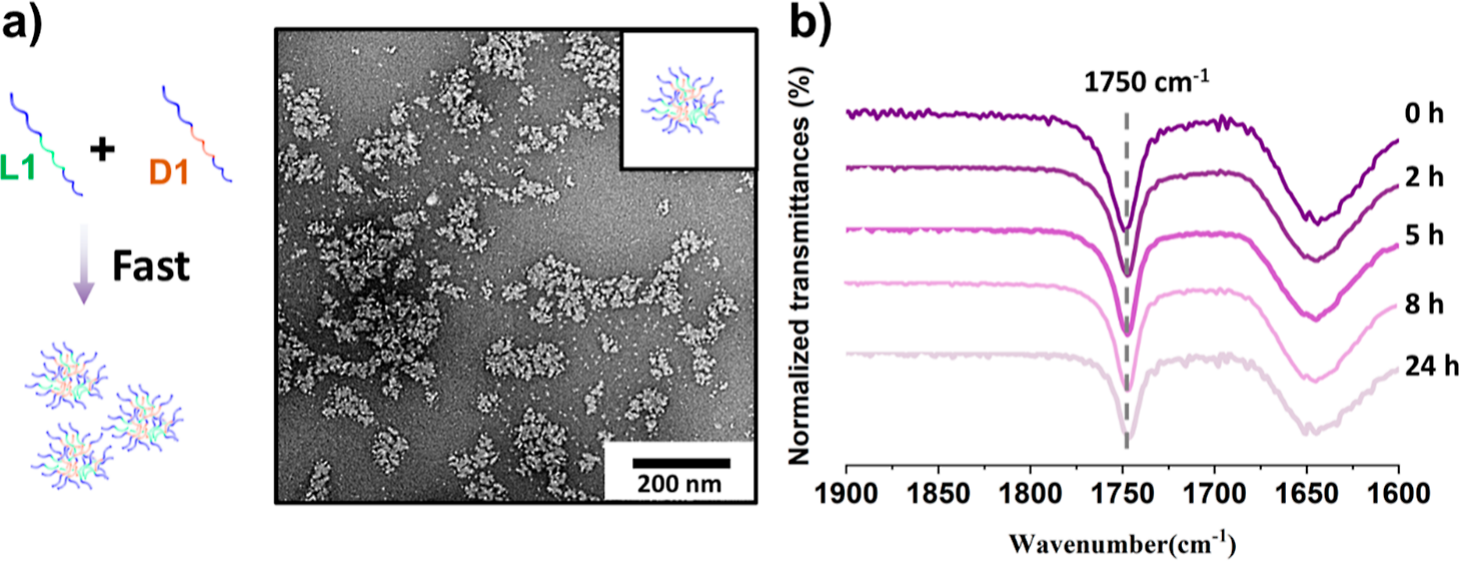
(a) Schematic representation of the formation of the new morphology driven by stereocomplexation and TEM micrographs of the aggregated worm-like structures obtained after mixing polymers **L1** and **D1** at body temperature (37 °C). The samples were negatively stained using uranyl acetate (0.5 wt %). (b) FTIR spectra revealed that the wavenumber of the carbonyl group vibration of PLA stays at 1750 cm^–1^ since the mixture of polymers **L1** and **D1**.

Previously, the SDMT using diblock copolymers was explained by considering the “unimer-exchange” mechanism in which the dynamic exchange of unimers between a free unimer and a bound unimer led to sufficient concentration of unimers in solution for stereocomplexation to occur.^32^ The enhanced stability of the stereocomplex crystallite then drives the equilibrium, until there are no homochiral micelles remaining. To further clarify that the “unimer-exchange” mechanism likely occurs in this system, a “unimer + micelles” experiment was carried out, i.e., copolymer **D1** and micelles **SA-L1** were mixed and aged in methanol at 37 °C (Figure S11). TEM and FTIR spectroscopic analyses of the morphologies over the duration of the experiment showed that the morphological transition was accomplished within 3 h, much quicker than that for the “micelles + micelles” (**SA-L1** + **SA-D1**, 24 h) analogue. This excluded the other possible pathway, i.e., “micelle fusion” to form the stereocomplex, where the transition of “unimer + micelles” should be slower in comparison to “micelles + micelles”. Finally, a “unimer + unimer” experiment was also undertaken; i.e., copolymers **L1** and **D1** were directly blended and aged together. In this case, the stereocomplex was formed immediately after mixing: the carbonyl vibration peak in the FTIR spectrum was 1746 cm^–1^, and TEM analysis did not reveal cylinders to be present (Figure 3a,b). These experiments further enable us to postulate that unimer disassembly is slow and indeed rate determining, relative to stereocomplexation. Interestingly, the morphologies formed by a directly blended stereocomplex and micelles + micelles were further compared by AFM (Figure S12), which showed consistent results with the TEM analysis; i.e., micelles + micelles stereocomplex assemblies are larger than their polymer/polymer counterpart. It is assumed that in the micelles + micelles experiment, few unimers in solution were formed, and the later released unimers might gradually grow on them, leading to larger features. But in the directly blended stereocomplex scenario, the coexistence of a large quantity of unimers results in many more seeds, when the growing steps could be suppressed, and thus, the forming assemblies are smaller. In conclusion, the balance of unimer versus assembly in a given solvent system is important to the stereocomplexation-driven transition occurring; more soluble unimers should lead to faster transition.

### Study of the Parameters Affecting the Morphological Transition

#### Effect of Core Length

**Figure 4:**
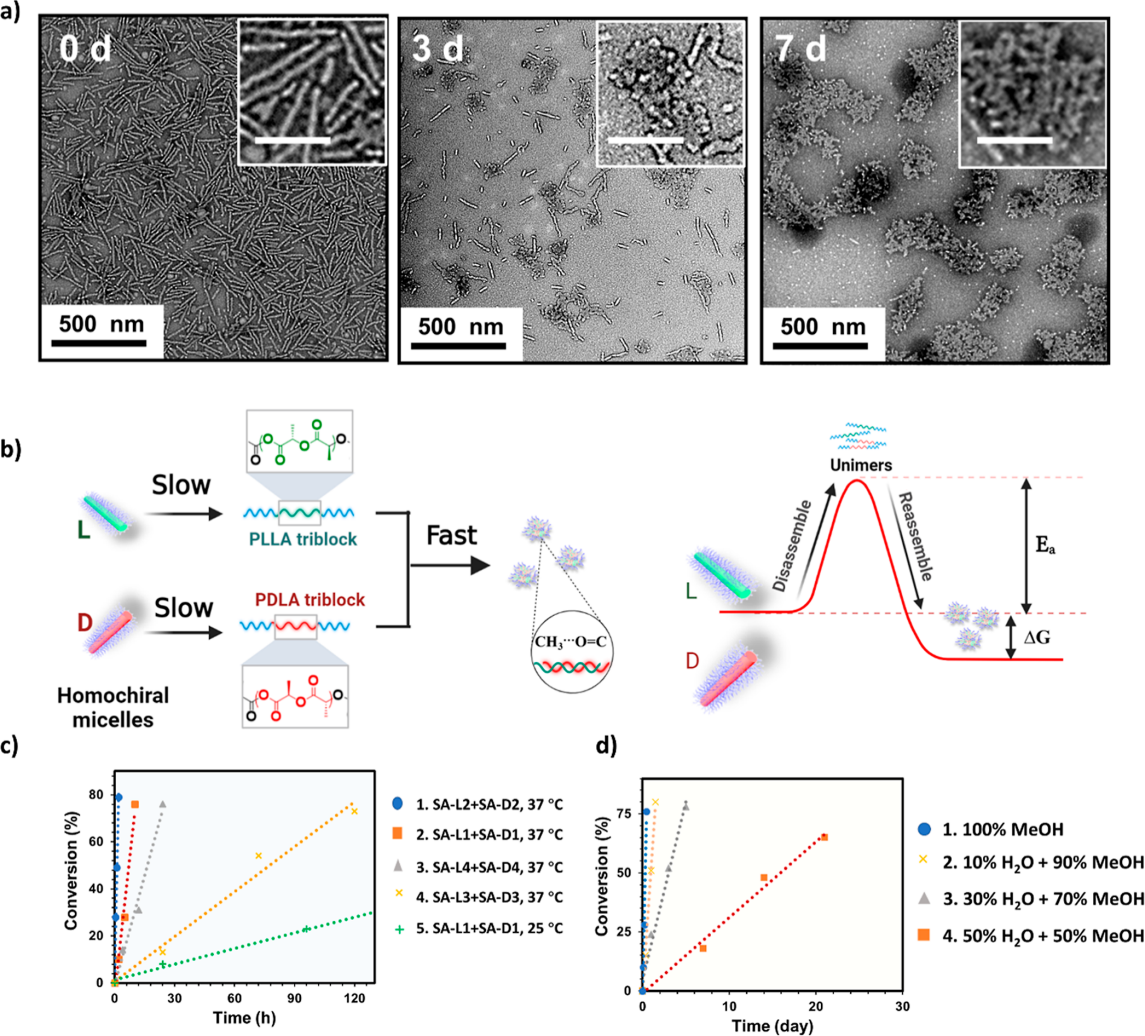
(a) TEM micrographs of the mixed assembly solutions **SA-L3** and **SA-D3** after aging at 37 °C in methanol for 0 h, 3 days, and 7 days. The samples were negatively stained using uranyl acetate (0.5 wt %). (b) Schematic representation of the proposed mechanism of SDMT of coil-rod-coil poly(lactic acid)-based cylindrical nanoassemblies. (c) Effect of core/corona lengths, corona chemistry, and temperature on the morphology transformation. The progression of morphological transition from enantiomer assemblies was determined by carbonyl vibration in IR. (1) Long Corona: PHEAAm_92_-*b*-PL(D)LA_32_-*b*-PHEAAm_92_, 37 °C; (2) standard, PHEAAm_42_-*b*-PL(D)LA_32_-*b*-PHEAAm_42_, 37 °C; (3) corona PDMA, PDMA_46_-*b*-PL(D)LA_32_-*b*-PDMA_46_, 37 °C; (4) long core, PHEAAm_65_-*b*-PL(D)LA_50_-*b*-PHEAAm_65_, 37 °C; and (5) standard, PHEAAm_42_-*b*-PL(D)LA_32_-*b*-PHEAAm_42_, 25 °C. (d) Influence of solvent property, i.e., methanol and water (MeOH/H_2_O) with different ratios for the morphological transition process of **SA-L1** and **SA-D1** determined by carbonyl vibration in IR.

Polymer block composition plays a vital role in unimer solubility; thus, polymers with different block compositions were expected to affect the transition speed in this system. The influence of the core block length was investigated first. To that end, the copolymers with long core length (i.e., **L3** and **D3**) were synthesized and assembled, while the block ratio (corona to core) was kept close to copolymers **L1** and **D1** (2:1). The assemblies (**SA-L3** and **SA-D3**), which were sonicated into shorter fibers before mixing them in MeOH (1 mg mL^–1^), were aged at 37 °C. Monitoring the samples by TEM (Figure 4a) revealed that the morphological transition was dramatically slowed down for the polymers that had longer PLA blocks, compared to that displayed by the copolymers with shorter core length values, i.e., **SA-L1** and **SA-D1** (Figure 4b). Indeed, lots of cylindrical features were still detectable on the grid after aging for 3 days. The FTIR spectra (Figures S13 and 4d) further support this observation since only a progression value of 55% was determined after 3 days of aging, while it took 7 days for 89% of homocrystalline PLA to convert to a stereocomplex (assessed by FTIR spectroscopy). It was reported that the core block length displayed a significant influence on the chain exchange kinetics of poly(styrene)-*b*-poly(ethylene-*alt*-propylene) (SEP) diblock copolymers, i.e., longer core results in a much slower exchanging rate.^37^ It is also believed that longer core length values slow down the unimer-assembly exchange rate in this system, thus delaying the formation of stereocomplex structures.

#### Effect of Corona Length/Chemistry

The effect of the corona block length values on the morphological transition was also studied at the same time. In particular, the hydrophilic block extended from DP = 84 to DP = 184 (**L2** and **D2**) for a fixed hydrophobic core PLA_32_. The corresponding assemblies **SA-L2** and **SA-D2** were sonicated in shorter micelles and were studied for their ability to undergo an SDMT. According to the data collected from TEM and FTIR spectroscopy (Figure S14), the transition process was significantly accelerated in comparison to that of the shorter corona counterparts, i.e., **SA-L1** and **SA-D1** (Figure 4b). Specifically, after aging for 3 h, stereoaggregates were the only features observed on the grid, while the quantification from the FTIR spectroscopic analysis determined a progression value of 100% at that time, which is much faster than the **SA-L1** and **SA-D1** counterpart (i.e., 5 h, 60%). From a solubility point of view, it was postulated that the increased solubility introduced by the longer corona length could lower the energy barrier to trigger unimer–micelle exchange, and thus, more frequent exchange is beneficial to speed up the morphological transition (Figure 4c). Other than the corona length, corona chemistry was also altered to dimethyl acrylamide to understand its influence on the transition. Polymers L4 and D4 are synthesized accordingly (Table 1) and assembled into **SA-L4** and **SA-D4**. Though the mixture of **SA-L4** and **SA-D4** still triggers the transition (Figure S15), it is much slower than SA-L1/D1 standard as shown in Figure 4c.

#### Effect of Aging Temperature

In addition to the block length values, the aging temperature was also inspected with respect to its effect on the morphological transition process. Cylindrical micelles **SA-L1** and **SA-D1** were aged at room temperature (25 °C) for comparison. The process was observed by TEM (Figure S16): fiber-like micelles were detected as the dominant shape on day 7, whereas until day 28, stereoaggregates were the only detectable features. Furthermore, the FTIR spectroscopic analysis revealed that the transition process was significantly slowed down at room temperature, with only 38 and 81% of progression toward stereocomplex being reached after 7 and 21 days of aging, respectively (Figures S16 and 4b). Not only does the lower temperature decelerate the “unimer-assembly” exchanging rate but also decreases the polymer solubility, which altogether slows down the transition to a great extent. This result exemplified the important role played by temperature on the kinetics of the morphological transition process.

#### Effect of Solvent

Since the SDMT of PHEAAm_*y*_-*b*-PL(D)LA_*x*_-*b*-PHEAAm_*y*_ assemblies had been well understood in methanol, the next step focused on investigating their performance in water considering their potential biotechnological applications. Hence, cylindrical micelles **SA-L1** and **SA-D1** were transferred to an aqueous solution by gradient dialysis. Equal amounts of them were mixed together in water (1 mg mL^–1^) and aged at body temperature (37 °C). After aging for 4 days, the fiber-like micelles were still prevalent in the assembly solution without any morphological transformation being observed (Figure S19c). The results were further consolidated by IR analysis, since almost all the C=O stretching vibration signals from the lactide unit stay in the homochiral state (wavenumber 1758 cm^–1^) (Figure S19d,e). This is because the solubility of copolymers **L1** and **D1** was much lower in water than in methanol, which prevented the extraction of the unimers from the assemblies. In other words, the energy barrier for the morphological transition in water was too high to induce the transformation in an aqueous solution. To further elucidate this assumption, the morphological transitions of assemblies **SA-L1** and **SA-D1** were also monitored in a mixed solvent methanol/water. As the ratio of water rose from 10% to 50%, the transformation was dramatically impeded (Figures S20 and 4d), consolidating the theory that polymer solubility is critical to the feasibility and speed of such transition.

## Conclusions

A series of well-defined homochiral cylindrical micelles based on PLA triblock copolymers were successfully fabricated by CDSA. The use of PLA stereocomplexation is to drive a morphological transition of the polymer micelles, without any external stimuli such as light or pH. Facilitated by a triblock copolymer design, the SDMT was mediated under mild conditions (methanol, body temperature). The investigation of the unimer-exchange mechanism revealed that the initial dissolution of the unimers from the homochiral assemblies was likely the rate determining step of this transition. Moreover, investigation of the molecular parameters of the block copolymers revealed that those that were more readily able to be solubilized in the transition media (longer corona-forming blocks, shorter core-forming blocks) resulted in more rapid morphological transitions. These results suggest that the design of polymer architectures with PLA blocks that can mediate a stereocomplex-driven morphological transition process should focus on those that are increasingly soluble in aqueous media under physiologically relevant conditions in order to enable the use of this unique morphological trigger in biotechnological applications.

## Supporting Information Available

Additional experimental details, characterization data, results and discussion, and supporting figures (PDF)
