# Supplementary material for: Stereocomplex-Driven Morphological Transition of Coil–Rod–Coil Poly(lactic acid)-Based Cylindrical Nanoparticles
Source: Macromolecules. 2023 Sep 25;56(19):7689–97. doi: 10.1021/acs.macromol.3c00653 (PMC10569100; doi:10.1021/acs.macromol.3c00653)
Supplement: Supplementary file 1 — ma3c00653_si_001.pdf [file ma3c00653_si_001.pdf]

Supplementary Information for

Stereocomplex-Driven Morphological Transition (SDMT) of Coil-rod-coil Poly(lactic acid)-Based Cylindrical Nanoparticles

Yujie Xie,[a, b, †] Wei Yu,[a, †] Tianlai Xia,[a] Rachel K. O'Reilly[a, *] and Andrew P. Dove[a, *]

a. School of Chemistry, University of Birmingham, Edgbaston, Birmingham, B15 2TT, United Kingdom.

b. School of Medicine, Shanghai University, Shanghai 200444, China.

† Authors contributed equally to this work.

Corresponding author:

r.oreilly@bham.ac.uk (ROR)

a.dove@bham.ac.uk (APD)

Experimental Section

Materials

Chemicals and solvents were purchased from Sigma-Aldrich, Acros, Fluka, TCI, Fisher Chemical, Alfa Aesar or VWR. L-Lactide was purchased from Corbion-Purac and recrystallized once from dichloromethane and twice from toluene. The monomer was dried over 3 Å molecular sieves for 3 days and recrystallized from toluene. 1,8-Diazabicyclo[5.4.0]undec-7-ene (DBU) and (-)-sparteine were distilled over CaH_2 before use.1-(3,5-Bis(trifluoromethyl)phenyl)-3-cyclohexyl-thiourea was prepared and purified as reported. 2,2′-Azobis(isobutyronitrile) (AIBN) was received from Molekula. After recrystallization from methanol, it was stored at 4 °C. Deuterated solvents were used as received from Apollo Scientific. Raft agent 2-(dodecylthiocarbonothioylthio)-2-methylpropionic acid (DDMAT) was synthesized following the procedure described in previous work. All the monomers used for polymerization went through basic aluminium oxide to remove the inhibitor.

Characterization techniques

Proton nuclear magnetic resonance (^1H NMR) spectra were recorded on a Bruker AV-400 spectrometer at 400 MHz. All spectra were recorded in d_6-DMSO unless otherwise specified. The chemical shifts were reported as δ in parts per million and quoted downfield from the internal standard tetramethylsilane (δ = 0 ppm).

Size exclusion chromatography (SEC) was performed using an Agilent 1260 Infinity Multi-Detector GPC System fitted with a refractive index and UV detectors and equipped with a guard column (Varian PLGel) and two PLGel 5 μm mixed-D columns. The mobile phase was DMF and 5 mM NH_4BF_4, at a flow rate of 1 mL min^{-1} at 50 °C. All data was analysed using

Cirrus v3.3 and Agilent GPC/SEC software v1, and the calibration curves were produced using Varian Polymer Laboratories linear PMMA standards.

Transmission electron microscopy (TEM) was performed using a JEOL 2100FX at 200 kV. TEM samples were prepared on a formvar/carbon film TEM grid. In short, 2 μL of sample solution (1 mg mL^{-1}) was deposited on the grid and left to air dry. 5 μL of uranyl acetate (UA, 1%) solution was then dropped on the grid for 60 seconds before blotting. The sample was kept in a desiccator overnight before characterization. TEM images were analysed by ImageJ software.

Wide Angle X-ray Scattering (WAXS) was performed on a Panalytical X'Pert Pro MPD equipped with a Cu Kα1 hybrid monochromator as the incident beam optics. Typically, freeze-dried particles (*ca.* 30 mg) were placed on a 10 mm sample holder, and standard "powder" 2θ–θ diffraction scans were carried out in the angular range from 10° to 30° 2θ at room temperature.

Fourier-transform infrared spectroscopy (FTIR) data were recorded (neat) on a Perkin Elmer Spectrum 100 FTIR Spectrometer. 5 μL assembly solution (5 mg mL^{-1}) was deposited on top of the detectors using attenuated total reflection to measure, the scan wavelength start from 1600 to 1900 cm^{-1}. The data was exported for analyse using Origin 2019. Deconvolution and curve fitting of the spectra in the region of 1780 to 1720 cm^{-1}. The fitting parameters 2θ–θ are listed below: Peak Type (Gaussian), Centre gravity (1750 cm^{-1}, 1760 cm^{-1}), FWHM (12 for 1750 cm^{-1} and 1760 cm^{-1}).

Material synthesis

Synthesis of poly(N-hydroxyethyl acrylamide)-block-poly(L(D)-lactide)-block-poly(N-hydroxyethyl acrylamide); (PHEAAm$_y$-b-PL(D)LA$_x$-b-PHEAAm$_y$)

Dual functionalized Macro-CTA was prepared according to previous work,[1] i.e. CTA-PLLA$_{32}$-CTA, (40.0 mg, 8.7 μmol), HEAAm (99.8 mg, 867.2 μmol) and AIBN (14.2 μL of a 10 mg mL^{-1} solution in DMSO) were dissolved in DMSO (0.5 mL) before transferring to a dried ampoule. After three freeze-pump-thaw cycles, the solution was sealed under argon and heated for 2 hours at 65 ℃ (70% conversion). The reaction was quenched in ice-cold bath and purified by precipitation into cold ethyl acetate (once) and cold diethyl ether (twice). The resultant pale yellow solid (yield 40%) was dried under vacuum: M_n, ^{1}H NMR = 14.4 kDa, DP = 84. M_n, SEC = 20.1 kDa, $Đ_M$ = 1.16. ^{1}H NMR (400 MHz, d_6-DMSO, 298 K): δ (ppm) 7.71 (br s, 1 H, NHCH$_2$CH$_2$), 5.16 (q, 2 H, $^3J_{H\text{-}H}$ = 6.9 Hz, OCH(CH3)CO), 4.96 (br s, 2 H, CH_2OH), 3.43 (br s, 2 H, CH$_2$CH_2OH), 1.46 (d, 3 H, $^3J_{H\text{-}H}$ = 7.1 Hz, OCH(CH_3)CO), 0.87 (t, 3 H, $^3J_{H\text{-}H}$ = 6.8 Hz, CH_3CH$_2$).

Crystallization-driven self-assembly method for PHEAAm$_y$-b-PL(D)LA$_x$-b-PHEAAm$_y$ block copolymers

All the triblock copolymers prepared in this paper were assembled in methanol as follows: the copolymer was dissolved in methanol at a concentration of 5 mg mL^{-1}, and the solution was stirred at room temperature (25 ℃) in a sealed vial for two days.

Sonication of PHEAAm$_y$-b-PL(D)LA$_x$-b-PHEAAm$_y$ cylindrical micelles

The cylindrical micelles derived from PHEAAm$_y$-*b*-PL(D)LA$_x$-*b*-PHEAAm$_y$ copolymers in methanol (5 mg mL^{-1}) were transformed into shorter fibres by probe sonication in an ice-cold bath for 4 minutes.

Material synthesis

The morphological transition speed is in a linear relationship to the time; therefore, the disassembly process could be a zero-order reaction. Applying the Arrhenius Equation, the activation energy of the disassembly is determined by the relationship between constant k over different temperatures. Taken SA-L1 and SA-D1 for study, and reaction rate constant at 25 ^{o}C, 37 ^{o}C and 45 ^{o}C are measured respectively. In the mixed solution, the assembly concentration is 2.5 g/L, when the M_n of polymer L1 is 14300 g/mol.

$K = \frac{dC}{dt} * conversion = \frac{2.5}{14300} * \frac{1}{3600} * slope$ (conversion vs time, unit is mol L^{-1} s^{-1})

According to Arrhenius equation:

$$\ln k = \frac{-Ea\,1}{R\quad T} + \ln A$$

The plot of lnk against $\frac{1}{T}$, (lnk, $\frac{1}{T}$) is achieved using the data acquired from the experiment:

Ea = 94.4 kJ/mol

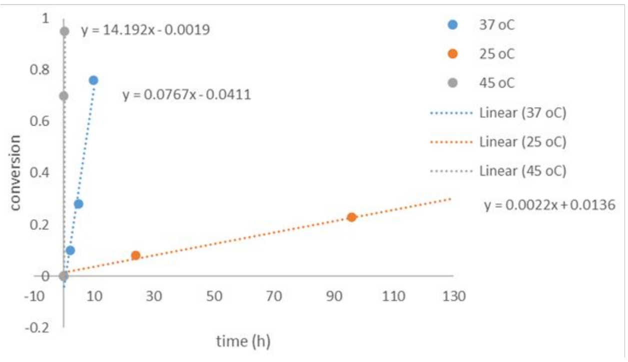

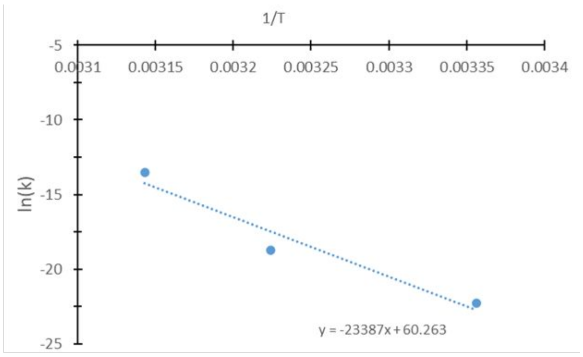

Supplementary figures

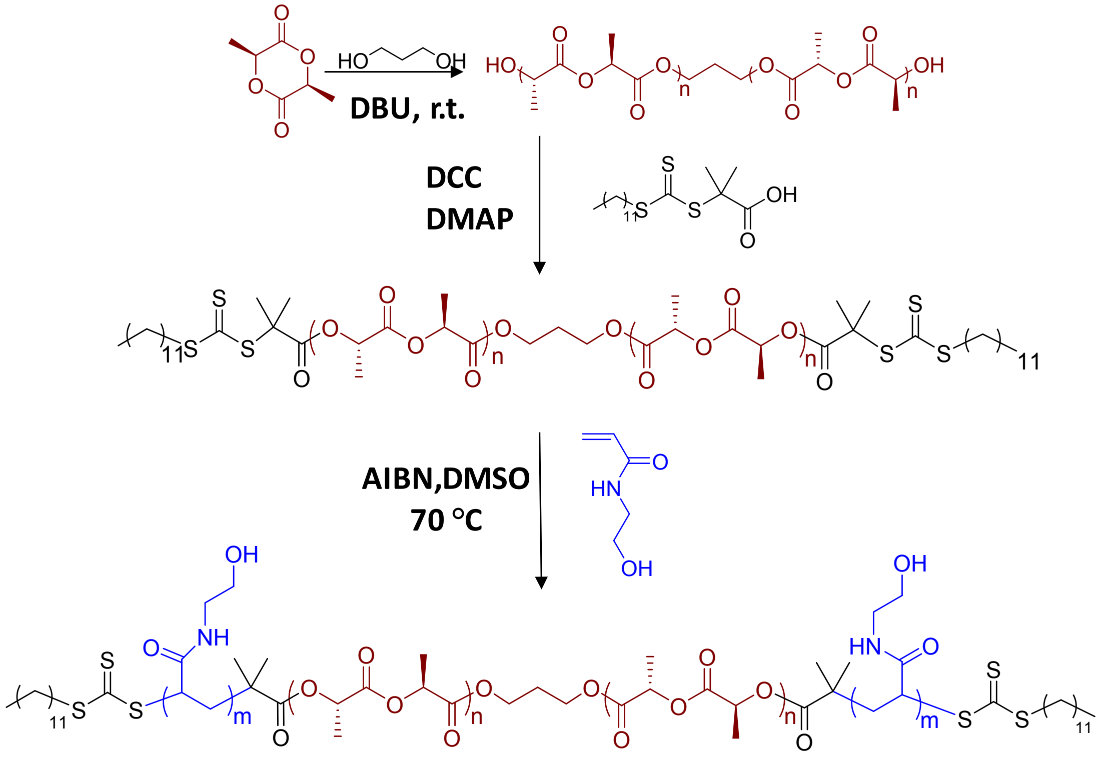

Scheme S1. Synthetic route for the preparation of PHEAAm-*b*-PLLA-*b*-PHEAAm.

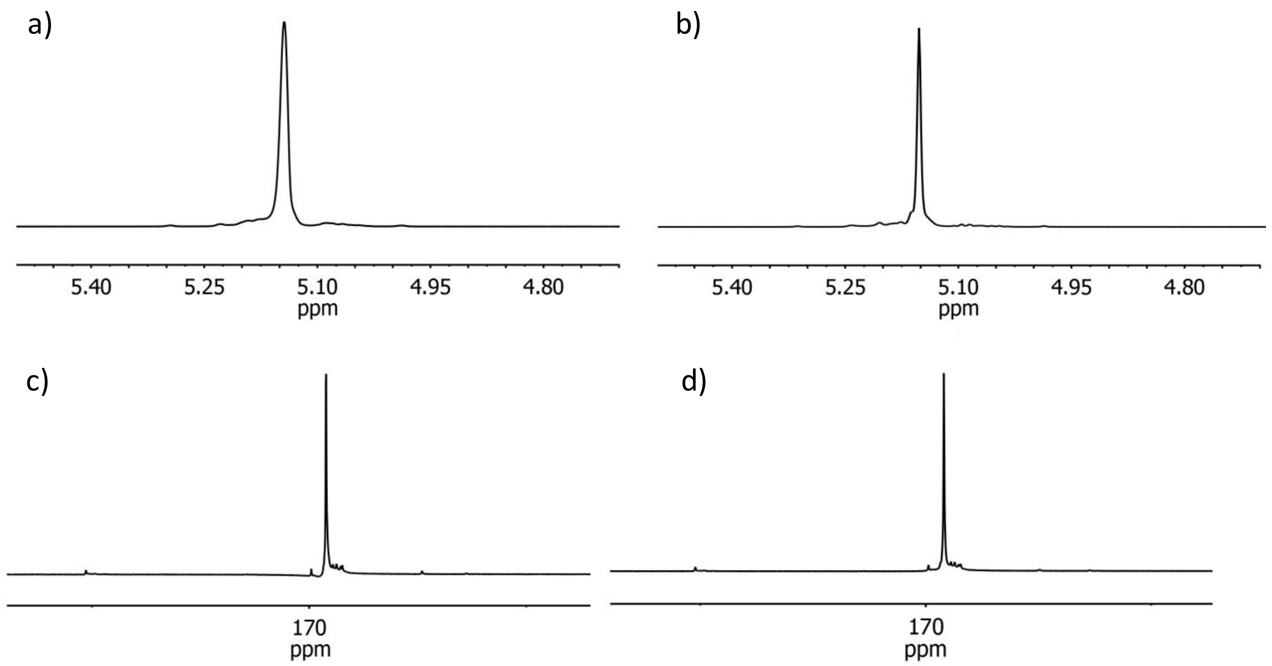

Figure S1. Homonuclear decoupled ^{1}H spectra of the methine region of (a) $PLLA_{32}$ and (b) $PDLA_{32}$ (500 MHz, $CDCl_3$). Quantitative ^{13}C NMR of (c) $PLLA_{32}$ and (d) $PDLA_{32}$.

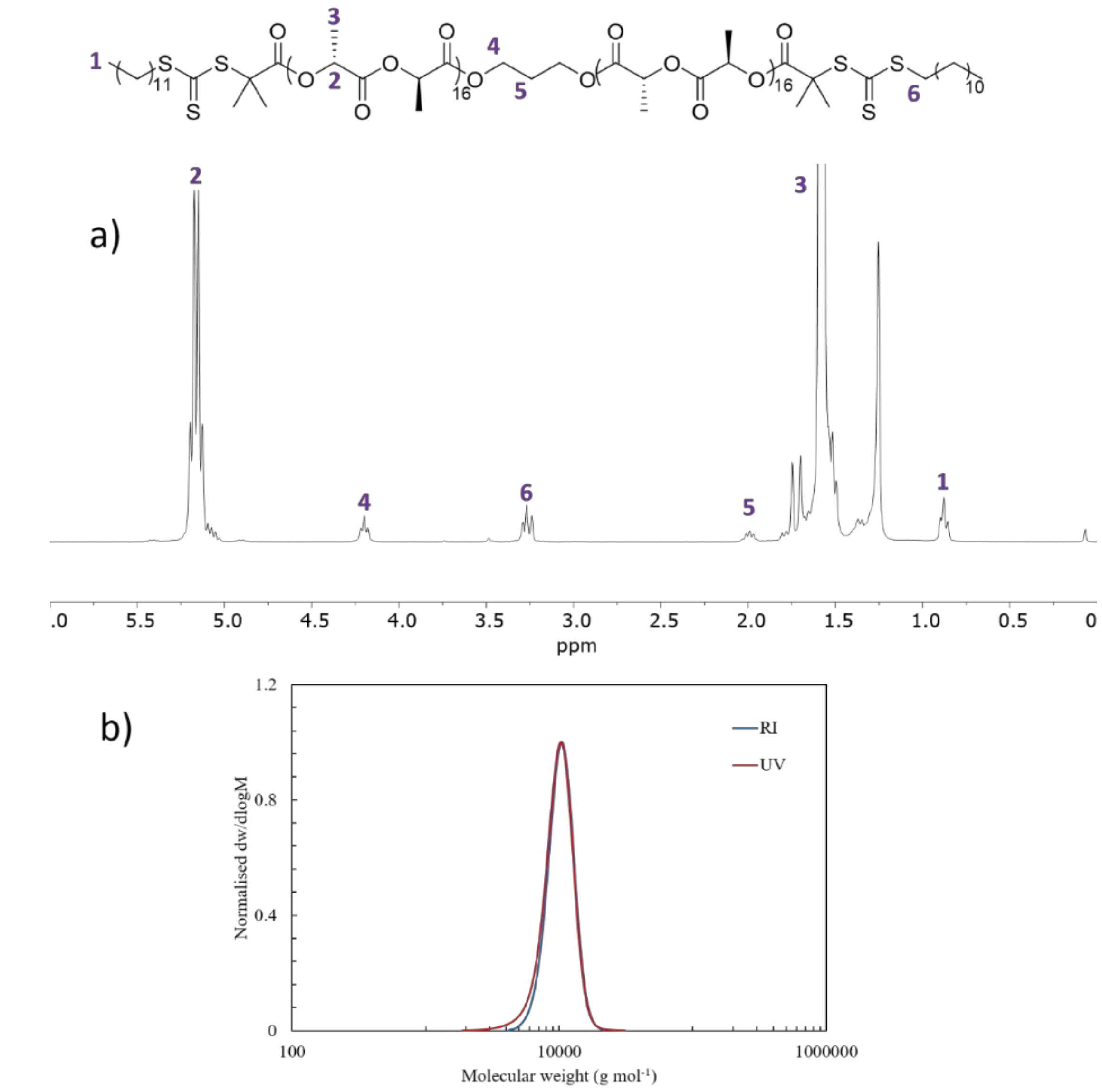

Figure S2. (a) ^{1}H NMR spectrum of CTA-PDLA$_{32}$-CTA (400 MHz, $CDCl_3$). (b) SEC analysis of CTA-PDLA$_{32}$-CTA (DMF with 5 mM NH_4BF_4).

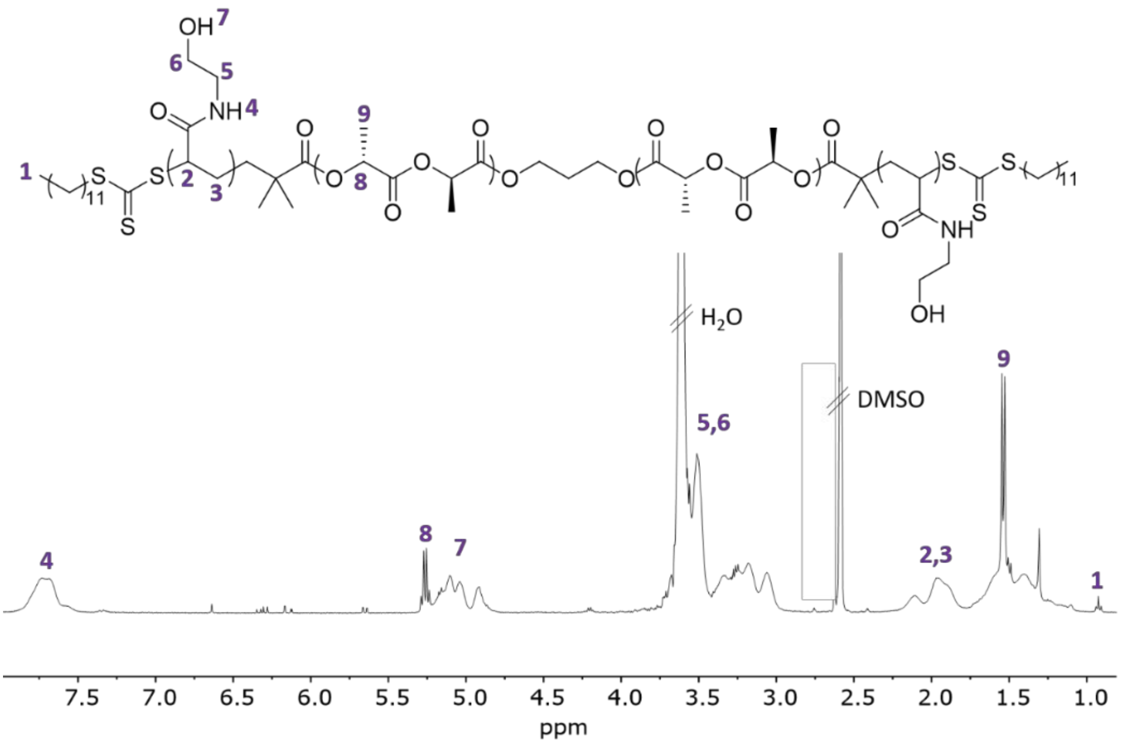

Figure S3. ^{1}H NMR spectrum (400 MHz, d_6-DMSO) of PHEAAm$_{92}$-*b*-PLLA$_{32}$-*b*-PHEAAm$_{92}$.

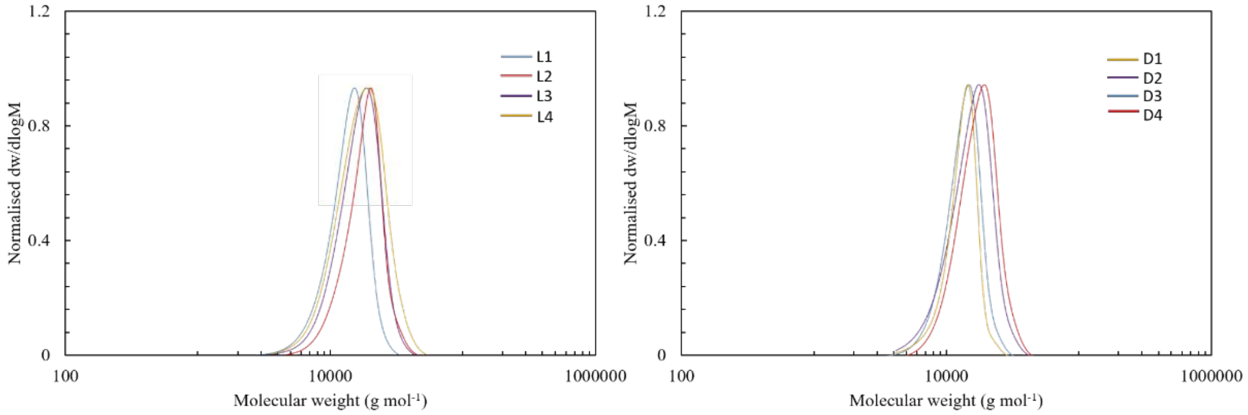

Figure S4. SEC traces of copolymers (a) **L1-L4** and (b) **D1-D4** from refractive index signals (DMF with 5 mM NH_4BF_4).

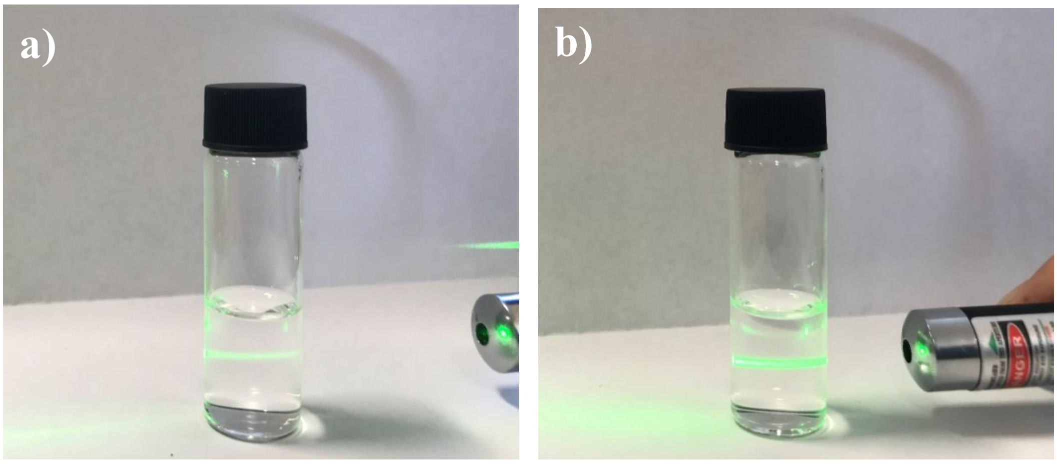

Figure S5. Triblock copolymer **L1** were dissolved and aged in methanol at a concentration of 5 mg mL^{-1} at room temperature. A laser pen was used to monitor the Tyndall effect of the triblock copolymer after aging for 1 h (a) and 24 h (b).

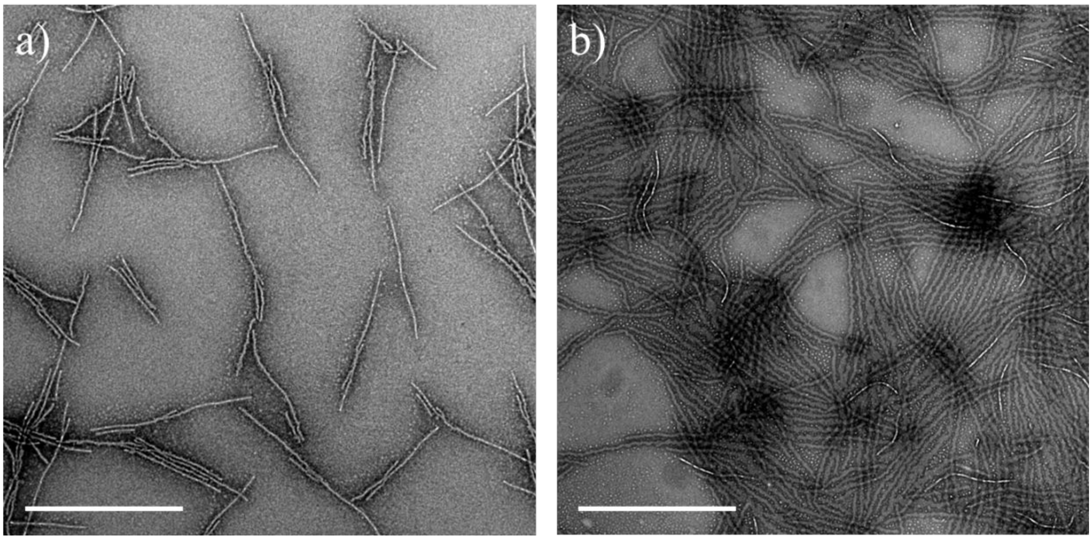

Figure S6. TEM micrographs of assemblies **SA-L1** and **SA-L2** after aging for one month. Samples were negatively stained using uranyl acetate (0.5 wt %). Scale bar = 1 μm.

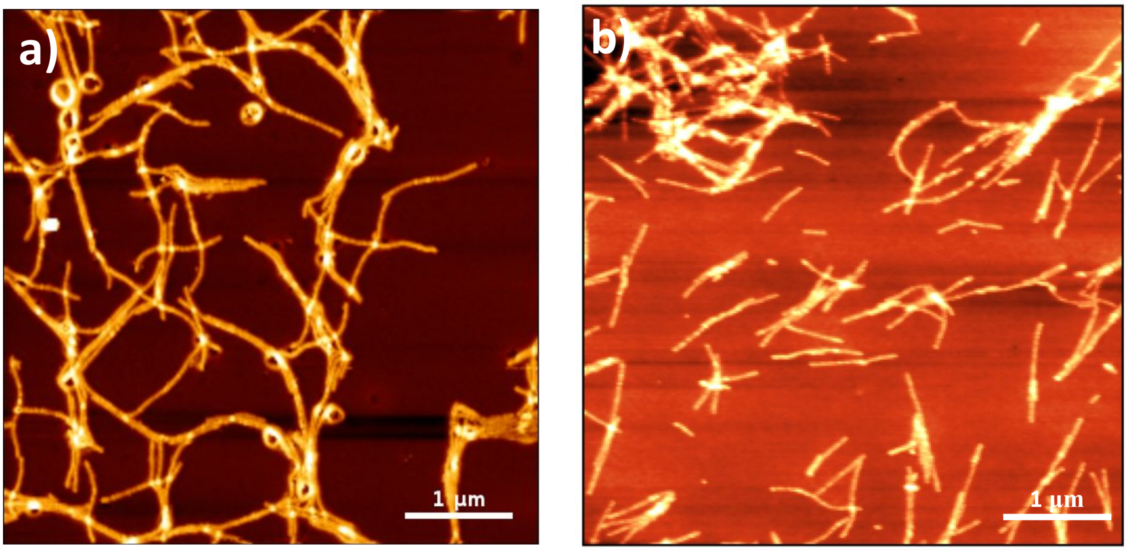

Figure S7. AFM images of triblock copolymer assemblies **SA-L1** (a) and **SA-D1** (b).

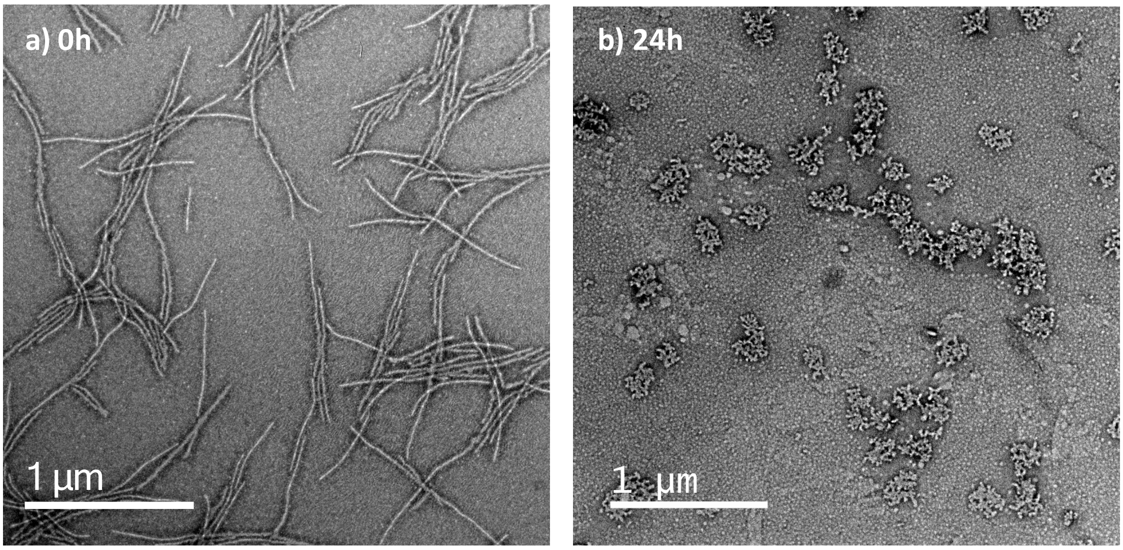

Figure S8. TEM micrographs of the mixed assembly solution **SA-L1** and **SA-D1** after aging at 37 ℃ for (a) 0 h and b) 24 h.

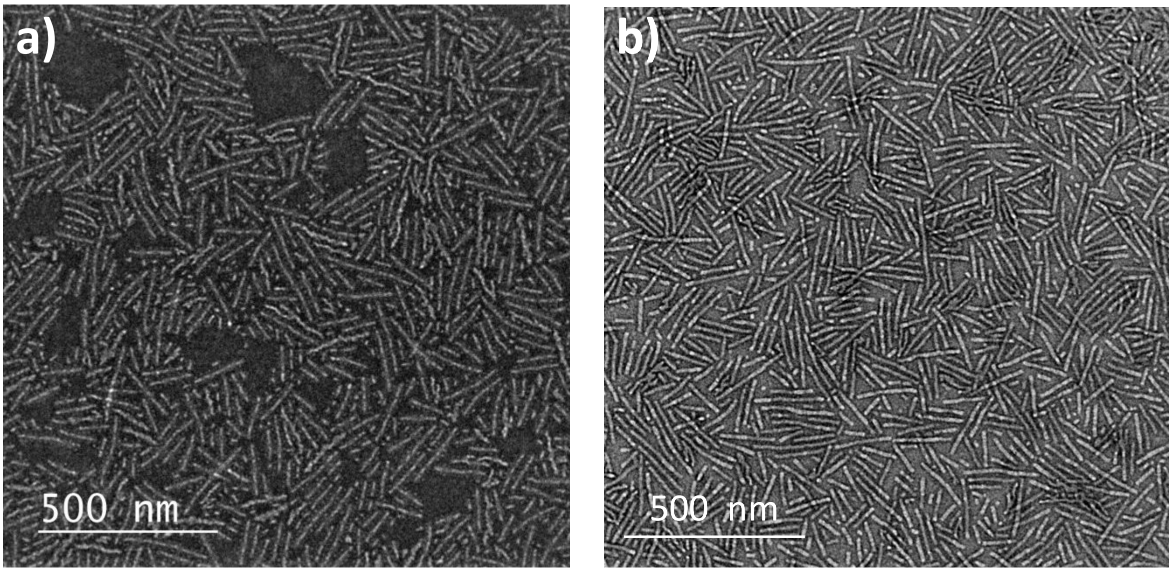

Figure S9. TEM micrographs of assemblies **SA-L1** (a) and **SA-D1** (b) after sonication at 0 °C using a sonic probe.

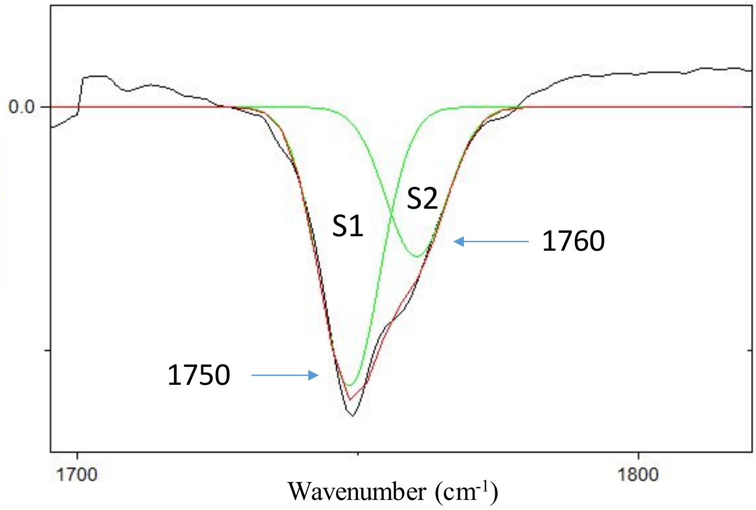

Figure S10. An example of the deconvolution procedure of IR data. The fitting parameters 2θ–θ are listed below: Peak Type (Gaussian), Centre gravity (1750 cm^{-1}, 1760 cm^{-1}), FWHM (12 for 1750 cm^{-1} and 1760 cm^{-1}). The red line represent the fitted curve, while the black line represent the measured spectrum. The green line represent curves of peak 1750 cm^{-1} and 1760 cm^{-1} after deconvolution.

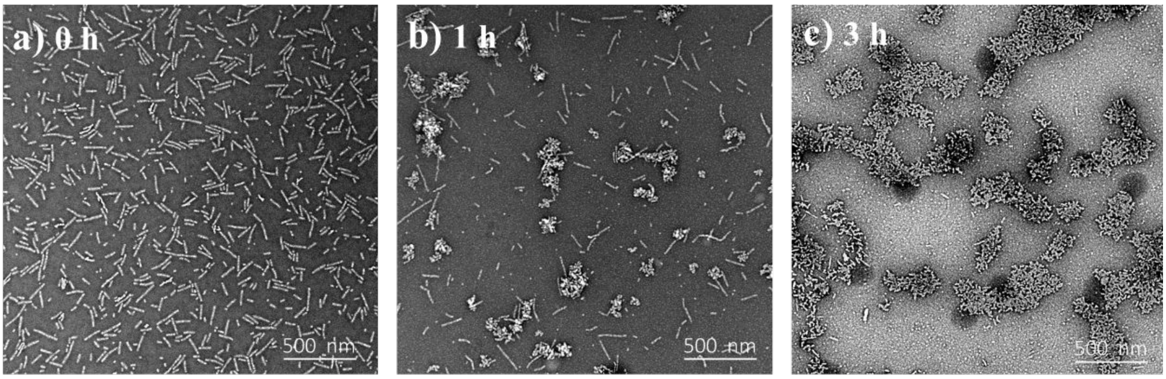

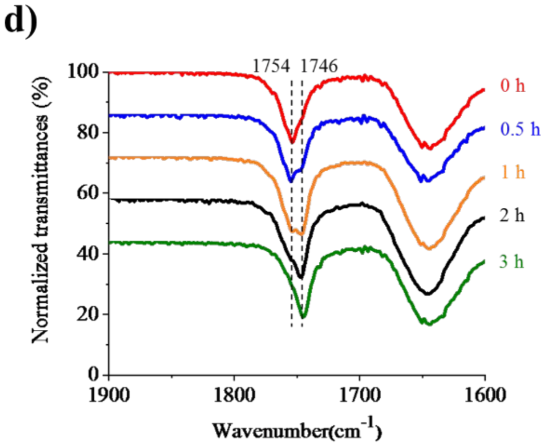

e)

Time (h)	Progression
0	0%
0.5	32%
1	65%
2	87%
3	100%

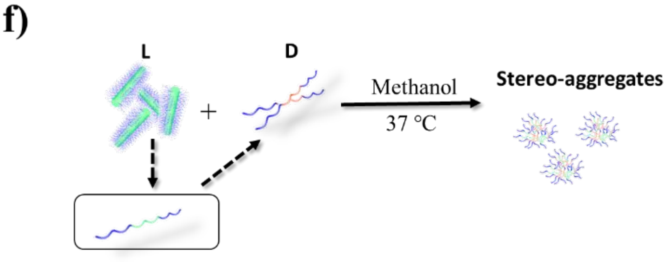

Figure S11. TEM micrographs of the mixed polymer **L3** and assembly **SA-D3** after aging at 37 °C in methanol for (a) 0 h, (b) 1 h, and (c) 3 h. Samples were negatively stained using uranyl acetate (0.5 wt %). (d,e) The progression of the morphological transition was monitored by FTIR spectra (f) Schematic representation of the formation of the new morphology triggered by stereocomplexation.

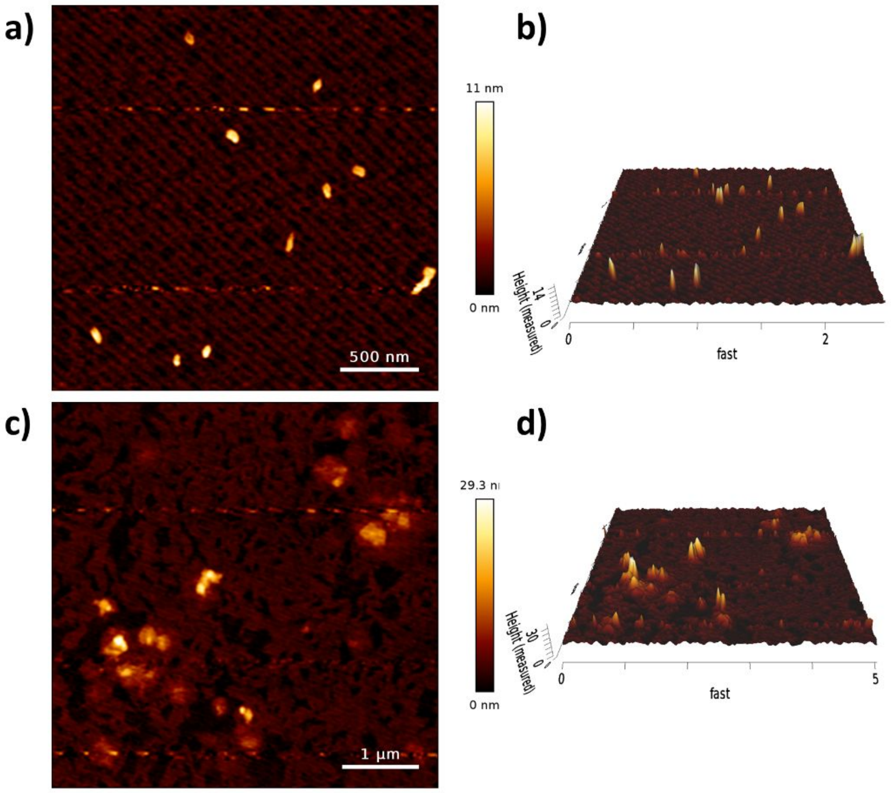

Figure S12 a-b) AFM image of the mixed unimer solution of L1 and D1 after aging at 37 °C for 24h in methanol; c-d) AFM image of the mixed assembly solution SA-L1 and SA-D1 after aging at 37 °C for 24h in methanol.

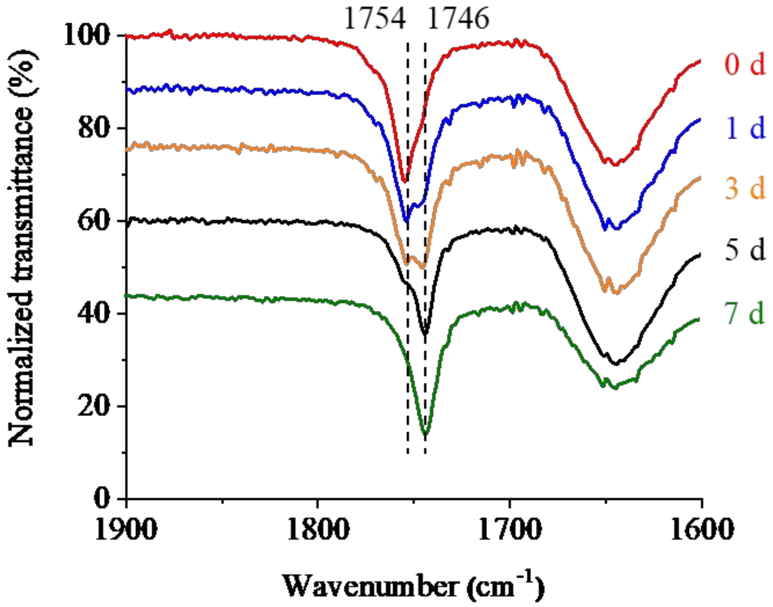

Figure S13. Mixed assembly solution SA-L3 and SA-D3 after aging at 37 ℃ in methanol. The progression of the morphological transition was monitored by FTIR spectra.

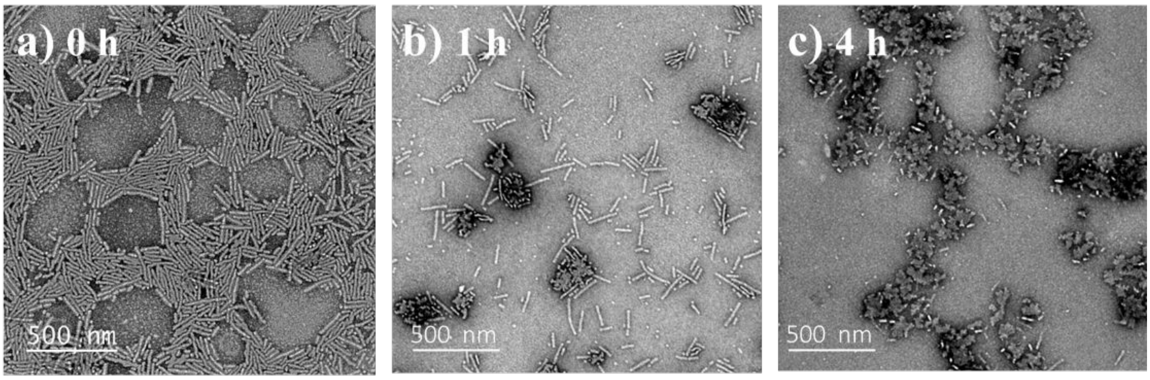

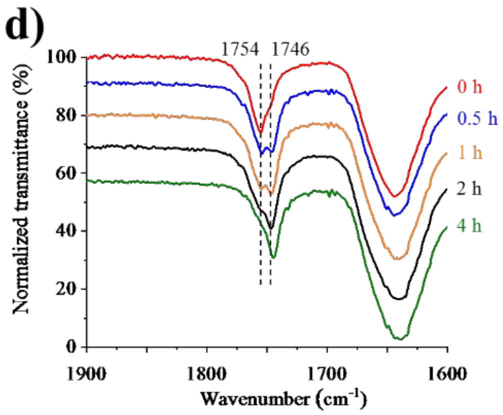

e)

Time (h)	Progression
0	0%
0.5	32%
1	65%
2	87%
3	100%

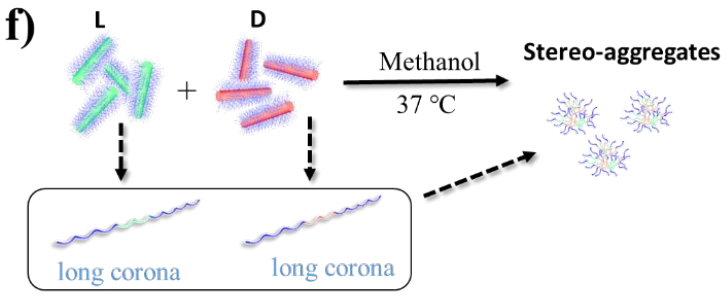

Figure S14. TEM micrographs of the mixed assembly **SA-L2** and assembly **SA-D2** after aging at 37 °C in methanol for (a) 0 h, (b) 1 h, and (c) 4 h. Samples were negatively stained using uranyl acetate (0.5 wt %). (d,e) The progression of the morphological transition was monitored by FTIR spectra (f) Schematic representation of the formation of the new morphology triggered by stereocomplexation.

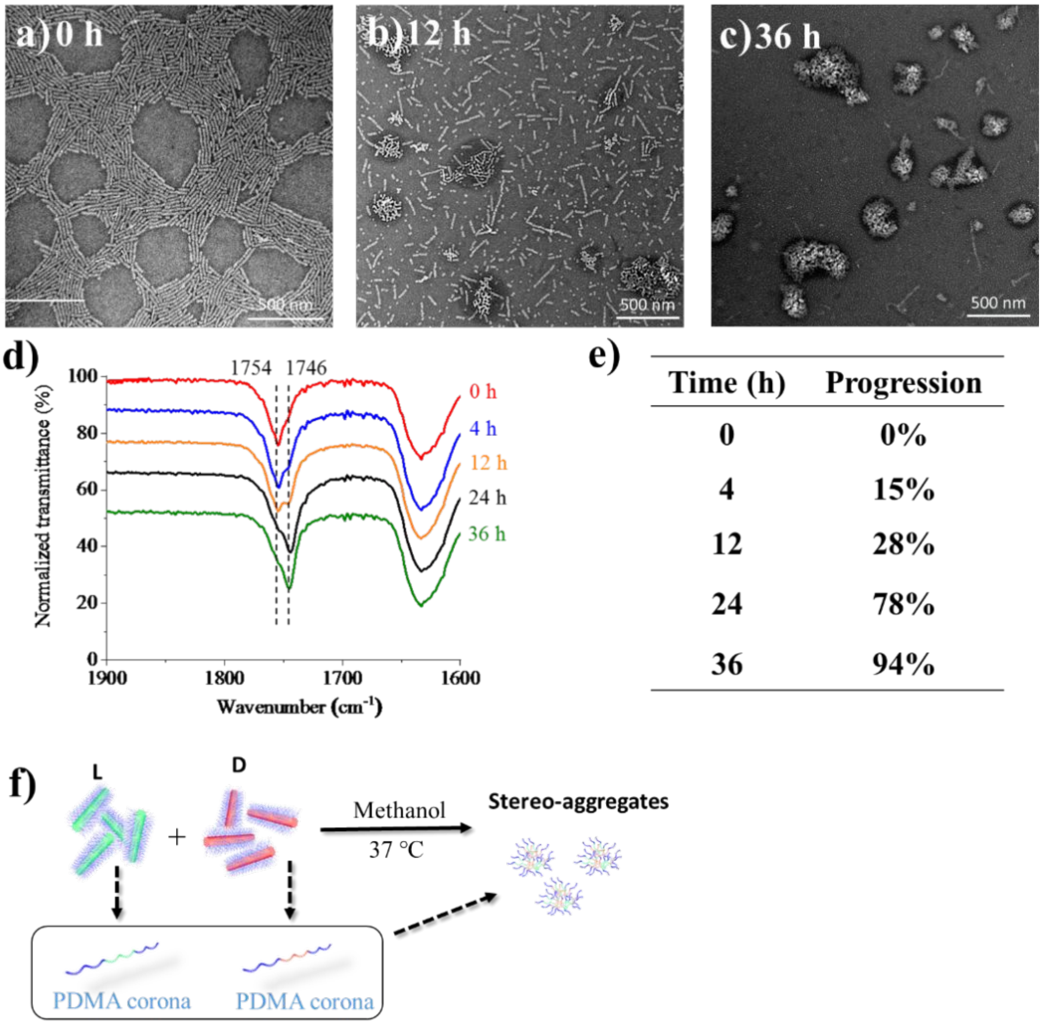

Time (h)	Progression
0	0%
4	15%
12	28%
24	78%
36	94%

Figure S15. TEM micrographs of the mixed assembly solution from polymer $PDMA_{45}$-*b*-$PLLA_{32}$-*b*-$PDMA_{45}$ and $PDMA_{50}$-*b*-$PDLA_{32}$-*b*-$PDMA_{50}$ after aging at body temperature (37 °C) in methanol for (a) 0 h, (b) 12 h, and (c) 36 h. Samples were negatively stained using uranyl acetate (0.5 wt %). Samples were negatively stained using uranyl acetate (0.5 wt %). (d,e) The progression of the morphological transition was monitored by FTIR spectra (f) Schematic representation of the formation of the new morphology triggered by sterecomplexation.

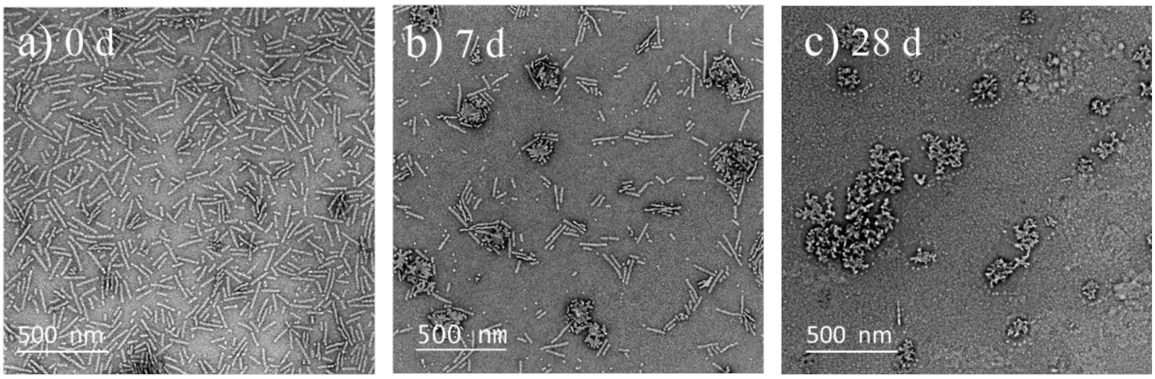

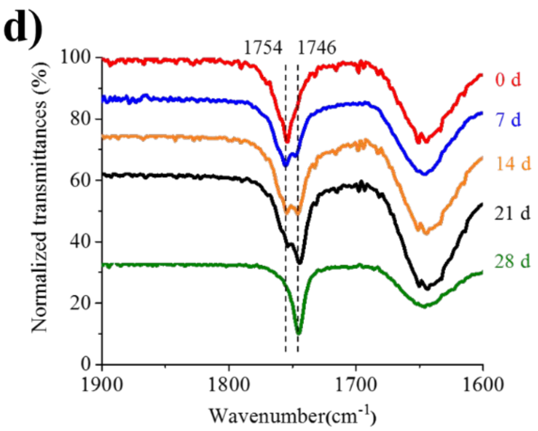

e)

Time (d)	Progression
**0**	**0%**
**7**	**38%**
**14**	**51%**
**21**	**81%**
**28**	**100%**

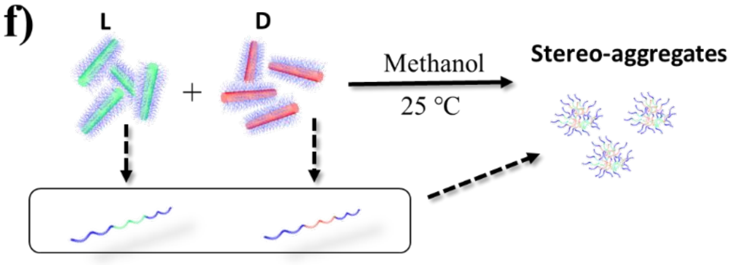

Figure S16. TEM micrographs of the mixed assembly solution **SA-L1** and **SA-D1** after aging at room temperature (25 ℃) in methanol for (a) 0 d, (b) 7 d, and (c) 28 d. Samples were negatively stained using uranyl acetate (0.5 wt %). Samples were negatively stained using uranyl acetate (0.5 wt %). (d,e) The progression of the morphological transition was monitored by FTIR spectra (f) Schematic representation of the formation of the new morphology triggered by stereocomplexation.

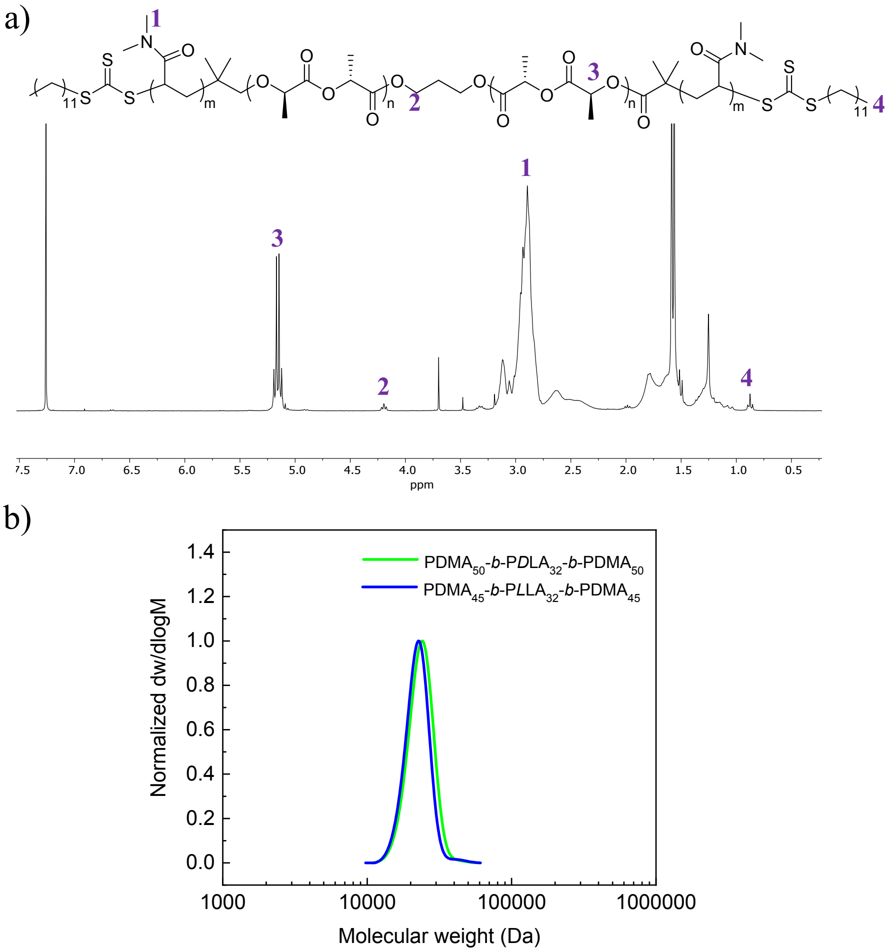

Figure S17. a) ^{1}H NMR spectrum of PDMA$_{45}$-*b*-PLLA$_{32}$-*b*-PDMA$_{45}$ triblock copolymer b) SEC curves of polymers PDMA$_{45}$-*b*-PLLA$_{32}$-*b*-PDMA$_{45}$ and PDMA$_{50}$-*b*-PDLA$_{32}$-*b*-PDMA$_{50}$ (DMF with 5 mM NH_4BF_4).

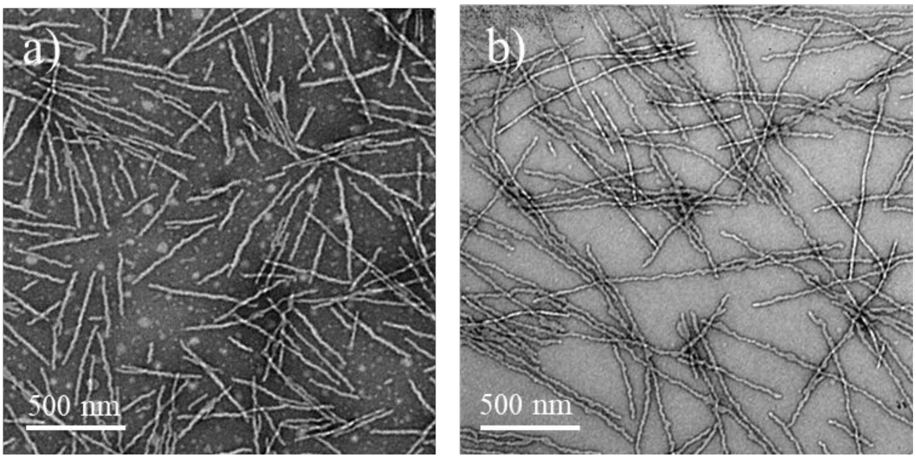

Figure S18. TEM micrographs of copolymers assembled in methanol 5 mg mL^{-1} at room temperature (25 °C) for two days: (a) PDMA$_{45}$-*b*-PLLA$_{32}$-*b*-PDMA$_{45}$ (b) PDMA$_{50}$-*b*-PDLA$_{32}$-*b*-PDMA$_{50}$. Samples were negatively stained using uranyl acetate (0.5 wt %). Scale bar = 500 nm.

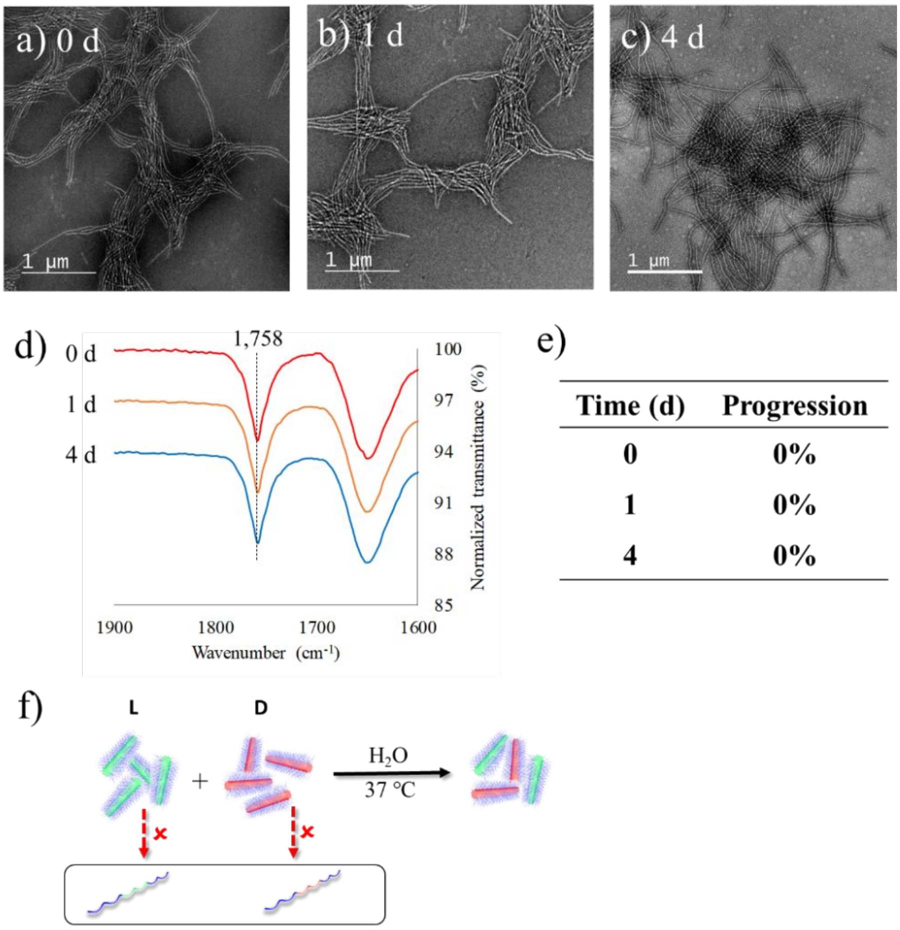

Time (d)	Progression
0	0%
1	0%
4	0%

Figure S19. TEM micrographs of the mixed assembly solution **SA-L1** and **SA-D1** after aging at 37 °C in water for (a) 0 d, (b) 1 d, and (c) 4 d. Samples were negatively stained using uranyl acetate (0.5 wt %). (d,e) The progression of the morphological transition was monitored by FTIR spectra (f) Schematic representation of the formation of the new morphology triggered by sterecomplexation.

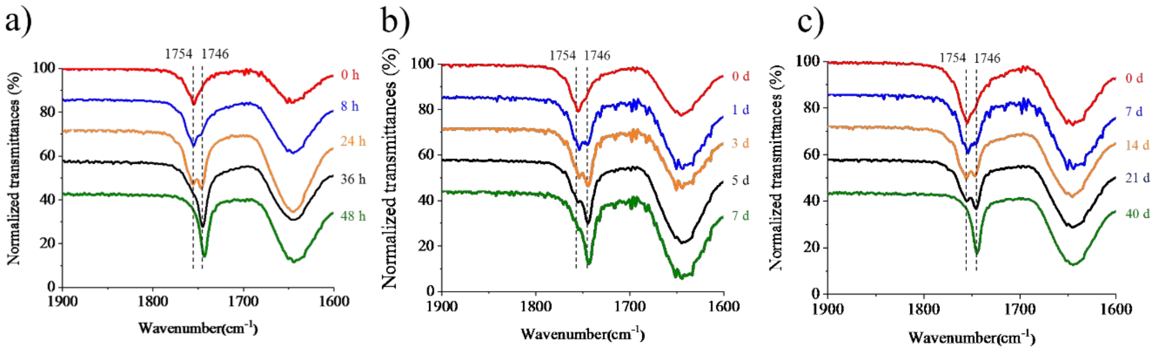

Figure S20. Assemblies **SA-L1** and **SA-D1** were added in mix solvent H_2O/MeOH and aged at 37 °C. The progression of the morphological transition was monitored by FTIR spectra, a) 10% H_2O + 90% MeOH b) 30% H_2O + 70% MeOH c) 50% H_2O + 50% MeOH.

References

1. Yu, W.; Inam, M.; Jones, J. R.; Dove, A. P.; O'Reilly, R. K., Understanding the CDSA of poly (lactide) containing triblock copolymers. *Polym. Chem.* **2017,** *8* (36), 5504-5512.
